# Dynamic transcriptional and chromatin accessibility landscape of medaka embryogenesis

**DOI:** 10.1101/gr.258871.119

**Published:** 2020-06

**Authors:** Yingshu Li, Yongjie Liu, Hang Yang, Ting Zhang, Kiyoshi Naruse, Qiang Tu

**Affiliations:** 1 State Key Laboratory of Molecular Developmental Biology, Institute of Genetics and Developmental Biology, Innovation Academy for Seed Design, Chinese Academy of Sciences, Beijing 100101, China;; 2 Key Laboratory of Genetic Network Biology, Institute of Genetics and Developmental Biology, Chinese Academy of Sciences, Beijing 100101, China;; 3 University of Chinese Academy of Sciences, Beijing 100049, China;; 4 Laboratory of Bioresources, National Institute for Basic Biology, Okazaki 444-8585, Aichi, Japan

## Abstract

Medaka (*Oryzias latipes*) has become an important vertebrate model widely used in genetics, developmental biology, environmental sciences, and many other fields. A high-quality genome sequence and a variety of genetic tools are available for this model organism. However, existing genome annotation is still rudimentary, as it was mainly based on computational prediction and short-read RNA-seq data. Here we report a dynamic transcriptome landscape of medaka embryogenesis profiled by long-read RNA-seq, short-read RNA-seq, and ATAC-seq. By integrating these data sets, we constructed a much-improved gene model set including about 17,000 novel isoforms and identified 1600 transcription factors, 1100 long noncoding RNAs, and 150,000 potential *cis*-regulatory elements as well. Time-series data sets provided another dimension of information. With the expression dynamics of genes and accessibility dynamics of *cis*-regulatory elements, we investigated isoform switching, as well as regulatory logic between accessible elements and genes, during embryogenesis. We built a user-friendly medaka omics data portal to present these data sets. This resource provides the first comprehensive omics data sets of medaka embryogenesis. Ultimately, we term these three assays as the minimum ENCODE toolbox and propose the use of it as the initial and essential profiling genomic assays for model organisms that have limited data available. This work will be of great value for the research community using medaka as the model organism and many others as well.

Medaka (*Oryzias latipes*) is a small freshwater fish native to East Asia, including Japan, Korea, and China. Compared with zebrafish, one of the most popular model organisms in life science, medaka has many unique characteristics, both genetic and physiological in nature. It is closely related to pufferfish, stickleback, and killifish, having diverged from a common ancestor with zebrafish ∼230 Myr ago (Kumar et al. 2017; Hughes et al. 2018). Medaka lives in small rivers, creeks, and rice paddies and can stand a wide range of temperatures (4°C–40°C) and salinities as shown by their tolerance to brackish water (Inoue and Takei 2002). Additionally, medaka has clearly defined sex chromosomes, and its sex determination mechanism has been intensively studied over the years (Matsuda et al. 2002). Consequently, various molecular, genetic, and developmental biology technologies have been developed for this model fish. More than 600 wild-type populations, mutants, transgenic, and inbred strains have been established and maintained at Japan National BioResource Project (NBRP) Medaka resource center. It has become an important vertebrate model widely used in genetics, developmental biology, endocrinology, biomedical science, and environmental sciences (Wittbrodt et al. 2002; Sasado et al. 2010; Kirchmaier et al. 2015).

The genome of medaka is ∼800 Mb, only half the size relative to that of the zebrafish genome. The genome sequence was published in 2007 with a contig N50 of 9.8 kb (Kasahara et al. 2007). Recently a high-quality (HQ) version based on the long-read single-molecule genome sequencing technology has been released (Ichikawa et al. 2017). The contig N50 is as long as 2.4 Mb for the Hd-rR strain, 1.3 Mb for the HNI-II strain, and 3.3 Mb for the HSOK strain (http://viewer.shigen.info/medaka/index.php). This version of the genome sequence of three inbred lines provides a solid foundation for various genomic analyses. However, past gene annotation efforts left room for improvement, as they were based primarily on computational prediction, comparative genomics, limited EST data, and short RNA-seq reads, all of which have inherent limitations. In addition, there is a lack of multiple omics data, further complicating the study of regulatory mechanisms.

To improve the quality of the existing reference genome annotation, we constructed data sets with multiple omics technologies and described a comprehensive landscape of transcriptional activity during medaka embryogenesis. We built a new gene model set, unveiled a large number of isoforms, and identified transcription factors (TFs) and long noncoding RNAs (lncRNAs) encoded in the genome; we obtained the temporal expression profiles of all these genes during embryogenesis and identified those that use different isoforms across developmental stages. Furthermore, we acquired chromatin accessibility dynamics and investigated *cis*- and *trans*-regulatory logic between accessible elements and genes. Ultimately, we built a user-friendly medaka omics data portal to help researchers explore the expression and regulatory information of genes of interest.

## Results

### Building multiple omics time-series data sets

**Figure 1.**
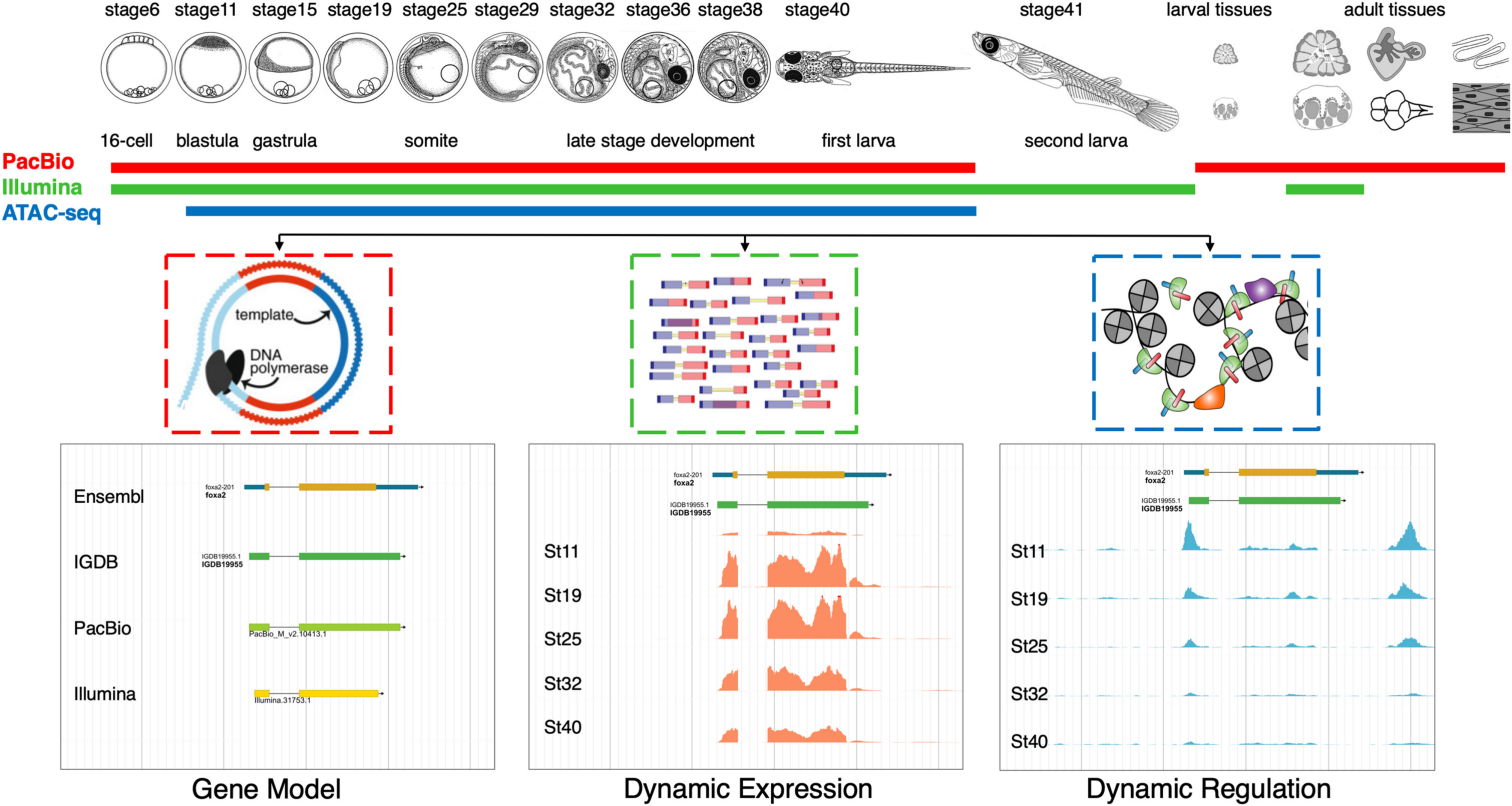
Overview of the study. Samples used are listed on the *top*, including embryonic stages and adult organs. Samples used for PacBio long-read sequencing (red bar) include 10 embryonic stages and six different adult organs. This data set was used for gene model construction. Samples used for Illumina short-read sequencing (green bar) include 10 embryonic stages, one larva stage, and two adult organs. This data set was used for gene expression quantification. Samples used for ATAC-seq (blue bar) contain nine embryonic stages. The resulted data set was used to define the genomic regulatory dynamics.

We surveyed samples of multiple embryonic stages with multiple omics technologies (Fig. 1). First, we collected 10 embryonic stages from cleavage, blastula, gastrula, somite, and late stages until hatching (9 d post fertilization). We also collected multiple larval and adult organ samples, including brain, heart, ovary, testis, gut, and muscle. In total, 19 samples were collected in this study (Supplemental Table S1). Next, 18 samples (all samples except a larval stage) were pooled into five libraries and sequenced using the single-molecule real-time (SMRT) platform from Pacific Biosciences (PacBio) to generate a long-read data set, which was used for gene model construction. Thirteen samples (embryonic stages, a larval stage, adult ovary/testis) were sequenced using the Illumina short-read sequencing platform, and this data set was used for gene expression quantification. Chromatin accessibility of nine embryonic samples was profiled using transposase-accessible chromatin with high-throughput sequencing (ATAC-seq) to identify potential regulatory elements (Buenrostro et al. 2013; Corces et al. 2017). All embryonic time points surveyed have two replicates. Most adult tissues were not profiled individually, so no further analysis was performed. In total, time-series data sets covering major embryonic stages were constructed by three different omics technologies.

### Gene model construction

As discussed above, the existing gene model set of medaka was generated mainly based on computational predictions including protein-to-genome alignments, annotation from reference species, and short-read RNA-sequencing data sets (https://www.ensembl.org/Oryzias_latipes/Info/Annotation). These methods have inherent limitations and could miss or yield inaccurate gene models. For example, short reads often fail in resolving large structural features, including alternative splicing, alternative transcription initiation, or alternative transcription termination. In addition, short reads originating from transcriptional noise and often mapping to intronic or intergenic regions may produce false gene models (Goodwin et al. 2016; Kuo et al. 2017; Nudelman et al. 2018). In contrast, with long-read RNA-sequencing technologies, it has become a reality that one read is one transcript (Wang et al. 2019). These technologies produce single reads covering the entire DNA or RNA molecules, independent of the error-prone computational assembly. Thus, these technologies could eliminate many common issues in data derived from short reads, and have become important tools to improve the gene annotation in multiple species (Sharon et al. 2013; Kuo et al. 2017; Zhang et al. 2017; Nudelman et al. 2018; Wang et al. 2018).

To generate an improved gene model set, we extracted HQ RNA from 18 samples, pooled and built five cDNA libraries, and then sequenced using the PacBio SMRT platform. In total, 30.5 million subreads were generated, which yielded 134,548 HQ full-length consensus transcripts. We mapped these consensus sequences to the latest version of the reference genome (Ichikawa et al. 2017). The mapping rate was 99.9%, which indicated that the quality of both transcriptome and genome is high. We merged mapped transcripts into a unified PacBio model set containing 17,523 genes with 28,427 transcripts.

**Figure 2.**
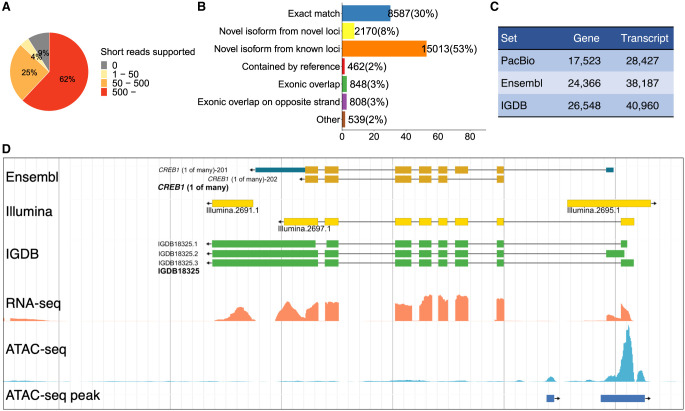
Gene model construction. (*A*) Splice junctions from the PacBio set validated by the Illumina short reads. Splice junctions are classified into four groups depending on the numbers of short reads supported. (*B*) Comparison of the PacBio set with the Ensembl set. The largest class is “novel isoforms from known loci.” (*C*) Numbers of genes and transcripts in three model sets. (*D*) An example showing the improved annotation of the medaka genome. The Ensembl model is correct overall. The Illumina models missed isoforms or are likely inaccurate owing to transcriptional noise. The IGDB model identified multiple novel isoforms with accurate boundaries. The ATAC-seq clearly showed the activity of the promoter.

Because we also generated short-read data sets for embryonic stages, we validated the quality of splicing junctions observed from the mapped full-length transcripts (Fig. 2A). We found more than half junctions (62%) were supported by over 500 short reads, and 29% were supported by one to 500 reads. Only 9% of junctions were not supported by short reads. These could be because biological samples used in long-read sequencing were more than those used in short-read sequencing. In addition, these nonsupported junctions could be true, as a previous study showed longer reads significantly improve the splice junction detection (Chhangawala et al. 2015). Overall, the strong support of splice junction from a different technology indicated the HQ of the PacBio long-read data set.

To estimate the differences between this set with the existing annotation in the Ensembl database, we compared the PacBio model set with the Ensembl model set (release 94) using the gffcompare program (Fig. 2B). Among 28,427 transcripts in the PacBio set, 8587 transcripts (30%) matches with the Ensembl set very well, which means models from two sets have the exact match of intron chain and the start/stop of the transcript may be slightly different. The majority of the PacBio set corresponds to novel transcripts, including 15,013 (53%) are novel isoforms from known loci, and 2170 (8%) are isoforms from novel loci. Therefore, this PacBio set discovered a large number of novel isoforms (17,183), providing a wealth of information on alternative splicing/initiation/termination in the medaka genome. The remaining 2657 (9%) transcripts are different from the Ensembl models in various ways. This low proportion of well-matched and the high proportion of novel isoforms were similar to a recent study using long-read RNA-seq technology to annotate the zebrafish genome (Nudelman et al. 2018), indicating that the short-read–based gene model construction is far insufficient to unveil the complex transcriptome structure.

On the other hand, the PacBio set covered fewer genes and transcripts. Of the 38,187 transcripts of the Ensembl set, 24,910 (65%) overlap with transcripts in the PacBio set (Supplemental Fig. S1A). As the throughput of long-read RNA-seq technologies is still limited and as samples surveyed in this study were also limited, those lowly or specifically expressed transcripts could be missed. By using the quantification data discussed below, we estimated the expression levels of PacBio-supported genes and Ensembl-only genes. The result showed that the latter, if they were true, were expressed at very low levels in surveyed samples (Supplemental Fig. S1B).

The PacBio set provides direct observation of full-length cDNAs but covers fewer loci, and the Ensembl set covers more loci but could generate false gene and transcript models. To take advantage of both, we used the PacBio set as the main source, supplemented by the Ensembl set. Roughly 61% of models came from the former, and 39% came from the latter (Fig. 2C). We named this combined version as the Institute of Genetics and Developmental Biology (IGDB) set and used it for further analysis. About 86% of splicing junctions in this set were supported by short reads (Supplemental Fig. S2). This IGDB set contains 26,548 genes and 40,960 transcripts. Figure 2D is an example showing the differences between different sets: The Ensembl model is correct as a whole, the IGDB model that was derived from the PacBio model provides accurate information on exon boundaries and alternative splicing events, and the Illumina model could be broken or with inaccurate exon boundaries. The median length of genes and transcripts are 7.2 kb and 2.5 kb. The median number of exons per transcript is eight (Supplemental Fig. S3). We transferred many types of functional annotation (Interpro protein domain matches, human/mouse/zebrafish/fly orthologs, Gene Ontology [GO] annotation) from the Ensembl database to the new IGDB gene set when possible. All sequences and functional annotations of the IGDB set can be retrieved using the medaka omics data portal discussed below.

### TF and lncRNA annotation

We paid particular attention to two classes of genes: TFs and lncRNAs.

TFs recognize specific DNA sequences, form a protein complex, and control the transcription of genes. Many TFs have in the past carried the moniker of “master regulators” or “selector genes” and play critical regulatory roles in all biological processes (Smith and Matthews 2016; Lambert et al. 2018). Identifying and classifying TFs will be of great value for all studies related to gene regulation. For example, a catalog of approximately 1600 human TFs had been made through extensive manual curation (Lambert et al. 2018). In this study, we used AnimalTFDB (Hu et al. 2019), a comprehensive database of animal TFs, to annotate TFs encoded in the medaka genome. In total, we identified 1646 TFs of 68 families (Supplemental Table S2). The largest three families are C2H2 zinc finger proteins (578, 35%), Homeobox (227, 14%), and the bHLH family (129, 8%) (Supplemental Fig. S4). They are also the largest three families encoded in the human genome.

LncRNAs are a heterogeneous class of RNAs longer than 200 nucleotides. They are often 5′-capped, spliced, and polyadenylated. However, they lack protein-coding potential and are not translated into proteins. The functions of the majority of lncRNAs are unknown, but some lncRNAs are known to play critical regulatory functions in diverse biological processes (Engreitz et al. 2016; Quinn and Chang 2016; Marchese et al. 2017). In the past decade, the total number of lncRNAs has grown rapidly as experimental and computational omics profiling technologies were improved substantially. However, many previous studies identified lncRNAs based on Illumina short reads, which has inherent difficulties of reconstructing transcript structures as discussed above. This particularly affects lncRNAs, as they often lack protein-coding capacity and conservation. Our PacBio long reads provide a solid base to identify lncRNAs expressed during medaka embryogenesis. We removed transcripts that either are short, are homologous to known coding genes, or have high coding potential. We noticed that known coding genes in the Ensembl database had high coding potential scores around one, whereas scores of novel genes showed a bimodal distribution, indicating that a large portion of them are noncoding genes (Supplemental Fig. S5). In the end, 1135 lncRNAs were identified (Supplemental Table S3). This work is a great expansion of lncRNA annotation in the medaka genome as no systematic survey had been performed yet. As expected, the majority of these lncRNAs are not conserved. We compared medaka lncRNAs with human and zebrafish lncRNAs collected in the RNAcentral database (The RNAcentral Consortium 2019). Only eight match with human lncRNAs, and 10 match with zebrafish lncRNA (*e*-value <10^−10^). LncRNAs are also expressed at relatively low levels compared with the protein-coding genes (Supplemental Fig. S6).

### Dynamic gene expression

Long-read RNA-sequencing technologies are powerful for elucidating transcript structure. However, the throughput of these technologies is not high enough to estimate the abundance of transcripts. Therefore, we used Illumina's short-read RNA-sequencing technology to profile gene expression at embryonic stages. In total, we generated 301 Gb (1020 million reads) of short-read data from two replicates, mapped these reads to the reference genome, and quantified the gene expression with our IGDB model set, in terms of transcripts per million (TPM) (Li et al. 2010). In the end, we obtained expression profiles of all genes during medaka embryogenesis, 79% (20,885) of which reach at least 1 TPM at one stage in both replicates. We calculated the Spearman's correlations among all stages (Supplemental Fig. S7) and also compared this data set with a previously published data set (Supplemental Fig. S8; Ichikawa et al. 2017). The high correlation between adjacent or similar stages indicated the HQ of the data.

**Figure 3.**
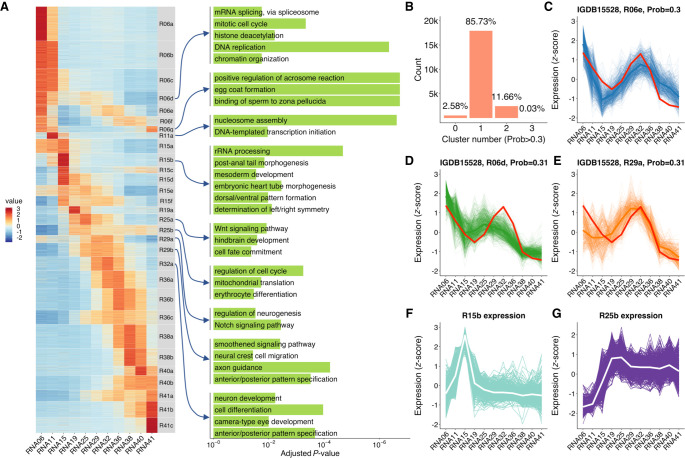
Clustering of expression profiles using a fuzzy clustering algorithm. (*A*) Heatmap of 30 expression profile clusters across embryogenesis. Genes expressed at >1 TPM in at least one stage are shown. Clusters were sorted by the stage when the maximum expression occurs. The bar plots on the *right* show selected GO enrichment of relevant clusters. (*B*) Adjustable fuzziness. With a cutoff of membership >0.3, 85.7% of genes were assigned to one cluster, 11.7% were assigned to two or more clusters, and 2.6% were not assigned to any cluster. (*C*–*E*) An example of one gene (*rnf19a*) assigned to three different clusters with membership >0.3, showing the similarity to multiple clusters. (*F*) Time-series expression profile of cluster R15b, which enriched mesoderm and body axis formation genes. The white line represents the average expression of the cluster, and the background lines represent all genes assigned to this cluster. (*G*) Time-series expression profile of cluster R25b, which enriched erythrocyte differentiation genes. The expression values were represented by TPM/*z*-score.

We clustered these expression profiles using the c-means fuzzy clustering method (Futschik and Carlisle 2005). Many clustering algorithms produce a hard partition of data, which means the given expression profile is assigned to just one cluster. The assumption of these algorithms is that the internal structure of the data is well separated. However, this is not true for gene expression profile clustering. The given expression profile could belong to multiple similar clusters with certain probabilities (Futschik and Carlisle 2005). The fuzzy clustering algorithm assigns each expression profile a membership value for each cluster so that researchers can obtain expression profiles belonging to a given cluster using different fuzziness values. By using Mfuzz (Kumar and Futschik 2007), we clustered expression profiles into 30 clusters (Fig. 3A). With a cutoff of membership >0.3, 85.7% of profiles were assigned to one cluster, 11.7% were assigned to two or more clusters, and 2.6% were not assigned to any cluster (Fig. 3B). Take gene *rnf19a* as an example; its expression profile is very similar to three clusters. Thus, its membership values for multiple clusters are all above 0.3 (Fig. 3C–E). Therefore, this fuzzy clustering method provides us an opportunity to retrieve all potential members of the cluster of interest by adjusting the membership cutoff.

GO enrichment analysis of these 30 clusters showed enrichment of a lot of functions corresponding to the relevant stages (Fig. 3A; Supplemental Fig. S9). For example, mesoderm developmental genes and body axis formation genes are highly enriched in cluster R15b, which reaches the peak expression level at stage 15, the mid-gastrula stage (Fig. 3F). Genes involved in the erythrocyte differentiation are enriched in cluster R25b, which reaches the peak expression level at stage 25 (Fig. 3G). This is the 18- to 19-somite stage, when the heart just starts to beat slowly and the blood begins circulating. The clustering information could be useful for identifying genes underlining major biological events and for understanding how these genes orchestrate in the complex embryogenesis.

### Isoform switching

As PacBio long reads provide single reads for alternatively spliced transcripts and as Illumina short reads provide quantification information of transcripts abundance, we are able to identify isoform switching events during medaka embryogenesis. We first looked for genes with multiple isoforms that have an expression level of TPM >1 for at least one stage, and we got 5313 genes that have multiple expressed isoforms (13,663). We defined isoform switching as that the predominant isoform (>60% of all isoforms of the given gene at the given stage) has been changed during embryogenesis, and we identified 1879 genes, which means 35% of genes with multiple expressed isoforms have switched the predominant transcripts during embryogenesis (Supplemental Table S4; Supplemental Fig. S10). By using the ATAC-seq data discussed below, potential *cis*-regulatory elements controlling the isoform switching could be identified (Supplemental Fig. S11).

**Figure 4.**
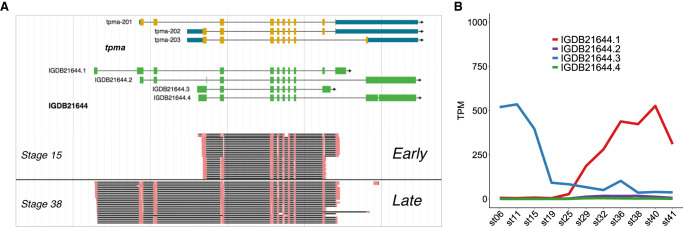
An isoform switching event during medaka embryogenesis. For gene *tpma*, the short isoform is predominant at early developmental stages, and the long isoform is so at late stages. (*A*) Genome view of reads mapped to different exons. (*B*) Expression dynamics of different isoforms.

Take *tpma* as an example; this gene encodes tropomyosin, which is a component of actin thin filament that constitutes myofibrils. Tropomyosin isoforms are known to be master regulators of the functions of actin filaments. These actin filaments play different functions in various physiological processes using different tropomyosin isoforms, including embryogenesis, morphogenesis, cell trafficking, cytokinesis, skeletal muscle, cancer, and so on (Gunning et al. 2015). Mammals have four tropomyosin genes, *TPM1*, *TPM2*, *TPM3*, and *TPM4*, which produce a total of 40 different isoforms (Geeves et al. 2015). Fish have six tropomyosin genes, including the paralogs of *TPM1* (*TPM1-1* and *TPM1-2*) and *TPM4* (*TPM4-1* and *TPM4-2*), and medaka *tpma* is the fish-specific paralog of *TPM1*. A previous study reported that several isoforms of zebrafish *tpma* are expressed in skeletal muscle (Dube et al. 2017). Here we not only identified multiple novel isoforms supported by both PacBio long reads and Illumina short reads but also found that the shorter isoform is predominant during early embryonic stages and that the longer isoform, which encodes an extra partial tropomyosin domain (Supplemental Fig. S12), is used during late stages (Fig. 4A,B). These isoforms switch at stage 19–25 when somites start to develop, which suggested that the long isoform might play an important role in somite development.

### Accessible element annotation

Active regulatory elements are often depleted for nucleosomes, accessible to the transcription machinery and also enzymes, so that they are sensitive to digestion by nucleases such as DNase I or Tn5 transposase. This feature enables the identification of regulatory elements with the assay for transposase-accessible chromatin using sequencing (ATAC-seq) (Thurman et al. 2012; Buenrostro et al. 2013). Identifying regulatory elements and characterizing their dynamics are critical to understand the regulatory mechanisms controlling the development. Previous studies have reported chromatin accessibility dynamics of early embryos of zebrafish, mouse, and human when the embryos are still relatively simple (Wu et al. 2016, 2018; Gao et al. 2018; Liu et al. 2018, 2019; Jung et al. 2019) or whole embryogenesis of *Caenorhabditis elegans* and amphioxus, but developmental stages surveyed were limited (Daugherty et al. 2017; Jänes et al. 2018; Marlétaz et al. 2018).

**Figure 5.**
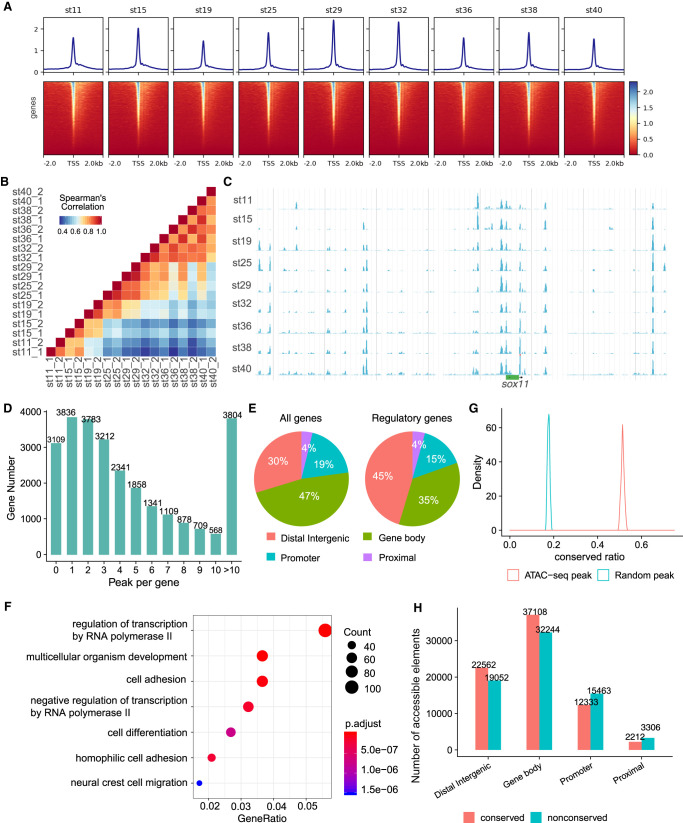
Overview of chromatin accessibility profiles. (*A*) Heatmaps of read distribution associated with genomic regions across all libraries. (*B*) Spearman's correlations among stages. (*C*) Representative screenshot of accessibility profiles across developmental stages. (*D*) Histogram of the number of accessible elements per gene. (*E*) Four types of elements according to their genomic locations. (*F*) GO enrichment analysis of genes with more distal intergenic accessible elements. (*G*) The distributions of conserved ratios of accessible elements and random regions. (*H*) The number of conserved or nonconserved accessible elements.

To profile the regulatory landscape of medaka embryogenesis, we applied ATAC-seq to detect genomic chromatin accessibility of nine embryonic stages. The percentage of mitochondrial reads and the enrichment of TSSs were used to determine the quality of ATAC-seq libraries (Corces et al. 2017). The read distribution associated with genomic regions showed that reads are highly enriched in the transcription start sites (TSSs) (Fig. 5A), which indicated the relatively low backgrounds in our profiles. The length of insert fragments showed a ∼200-bp period and a clear periodicity equal to the helical pitch of DNA (∼10.5 bp) (Supplemental Fig. S13; Buenrostro et al. 2013). ATAC-seq data from two replicates and two adjacent time points showed high correlations, indicating that ATAC-seq can reliably and reproducibly measure chromatin accessibility in these samples (Fig. 5B). A high correlation was also found between our data and a previously published smaller data set (Supplemental Fig. S14; Marlétaz et al. 2018).

To define DNA elements that are accessible at any developmental stage, we identified all peaks of significant ATAC-seq-read enrichment and merged them across all developmental samples. To build a HQ data set, we used a stringent filter (−log(*q*-value) > 7) to keep only highly confident peaks (Fig. 5C). Eventually, we obtained 149,539 accessible elements. They were assigned to the closest genes, and ∼88% of genes (23,439) have at least one highly confident accessible element (Fig. 5D). Across all developmental stages surveyed, 90% of elements (135,516) show variable accessibility for over twofold change, and 40% of elements (60,260) show variable accessibility for over fivefold change. These observations suggested a very dynamic landscape of DNA accessibility during medaka embryogenesis.

We classified these elements into four categories according to their genomic locations, namely, the distance to the nearest genes (Fig. 5E). We found that for all genes, 47% of elements are enriched in gene-body regions, 19% are enriched in promoter regions, and 30% are enriched distal intergenic regions. Genes associated with the GO term “regulation of transcription” have far more distal elements (45% vs. 30%) (Fig. 5E; Supplemental Fig. S15). An extreme example is *sox11*, which has a large number of accessible elements, but no neighboring gene exists within ∼350 kb (Fig. 5C). GO analysis also showed that genes with multiple (more than two) distal elements are highly enriched in the term “regulation of transcription” (Fig. 5F). Further studies using ATAC-seq data from human (Wu et al. 2018), mouse, chicken (Uesaka et al. 2019), and zebrafish (Liu et al. 2018) also showed this term enrichment in genes with multiple (more than two) distal elements (Supplemental Fig. S16). These indicated that regulatory genes have a more complex regulatory apparatus.

To examine the evolutionary conservation of accessible elements, we analyzed the selective constraint of these elements. First, we downloaded constrained regions of the medaka genome computed based on 51 fish genomes from the Ensembl Compara database. Accessible elements that overlapped with constrained regions were defined as conserved, and 51% elements fall into this category. We randomly sampled genomic elements with a similar length distribution to accessible elements, and only 18% of them were conserved. We repeated the sampling process 200 times and found the conserved ratio of accessible elements is significantly higher than that of random peaks (Kolmogorov–Smirnov test, *P*-value = 2.2 × 10^−16^) (Fig. 5G). This indicated that accessible elements are under higher selective constraint than expected. We found that among the four different categories of accessible elements, distal intergenic and gene-body elements are more conserved than others (Fig. 5H). Again, genes with more conserved elements than nonconserved elements (more than twofold) are enriched in the GO term “regulation of transcription” (Supplemental Fig. S17).

### *Cis*-regulatory logic

One of the major difficulties in accessible element analysis is how to link these elements to the genes they regulate. The common practice is to assign the elements to their closest genes, assuming a correlation between functional regulation and genomic distance. Previous studies had shown that the “closest gene” assignment is likely correct 90% of the time (Yoshida et al. 2019). Therefore, we used the genomic location as the rough proxy to establish the link between the accessible elements and genes.

**Figure 6.**
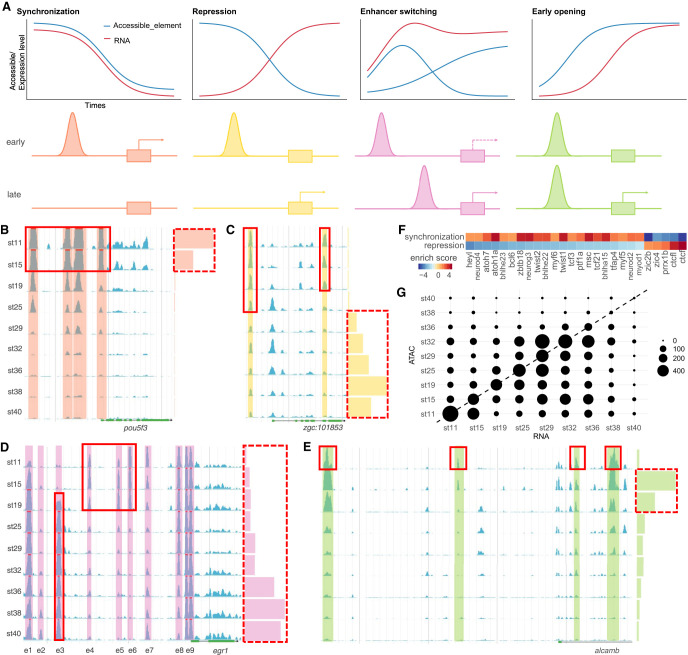
*Cis*-regulatory logic between genes and accessible elements. (*A*) Schematic diagram of four common types of *cis*-regulatory logic: synchronization, repression, enhancer switching, and early opening. (*B*–*E*) Cases of four types of *cis*-regulatory logic. Chromatin accessibility is presented by blue peaks with color shading. Gene expression is presented by color bars on the *right*. (*B*) Synchronization. (*C*) Repression. (*D*) Enhancer switching. (*E*) Early opening. (*F*) Differential TF binding motif enrichments between synchronization and repression peaks. (*G*) Early opening events are common. Circle position denotes the stage when gene expression first reaches 50% of its maximum (*x*-axis) versus the stage when the accessibility of the promoter first reaches 50% of its maximum (*y*-axis). The circle size indicates the number of genes.

To uncover the *cis*-regulatory logic between genes and accessible elements, we first assigned elements to its nearest gene and then calculated the correlation between the transcriptional activity of genes and the accessibility of elements. We found at least four common types of *cis*-regulatory logic: synchronization, repression, enhancer switching, and early opening (Fig. 6A–E).

The first type is synchronization. For example, there are four upstream accessible elements near *pou5f3*, and the accessibility of all these elements and the promoter as well are highly synchronized with the expression of *pou5f3* (Fig. 6B). The precise synchronization indicated that four enhancer elements and the promoter control the timing of *pou5f3* expression during embryogenesis. When using Spearman's correlation >0.8 as the cutoff, there are 7839 elements that show tight synchronization between their accessibility and the expression of the corresponding 4077 genes.

The second type is repression. An upstream accessible element of *zgc:101853* might be a repression element because the expression of *zgc:101853* and the accessibility of this element are mutually exclusive during embryogenesis (Fig. 6C). Similarly, when using a Spearman's correlation of less than −0.8 as the cutoff, there are 3396 elements that show repression regulatory logic with their corresponding 2533 genes. We then asked whether synchronization and repression accessible elements are regulated by different TFs. We used GimmeMotifs (Bruse and van Heeringen 2018) to identify differential TF binding motifs between two types of elements and found that CTCF and CTCFL binding motifs are highly enriched in repression accessible elements (Fig. 6F).

The third type is enhancer switching, a more complex scenario. For example, the gene *egr1* has eight accessible elements (Fig. 6D). It is expressed at relatively low levels (∼10% of the max level) at the early stages and then gradually turned on, reaching high levels (∼65%–100% of the max level) from stage 36 to 38. At very early stages (stage-11 blastula to stage-19 two-somite), elements e2 and e3 are closed, whereas e4 to e6 are open. Along with the boost of the transcription activity, e2 and e3 gradually become more accessible, whereas e4 to e6 are turned off. The promoter element e9 is constitutively open, and e1 is similar to e9 except that it is almost closed at stage 11. These observations indicated that e2 and e3 probably control the late-phase high-level expression, and e4 to e6 contribute to the early-phase low-level expression. The enhancers switch at different developmental stages. The promoter e9, and element e1 as well, might be necessary for the transcription activity of *egr1* and does not contribute to the switch of different expression phases. A previous study of zebrafish had shown that the expression of *egr1* is first detected in the presomitic mesoderm at early stages. Then, its expression is observed only in specific brain areas. Eventually, *egr1* is expressed in specific domains of multiple tissues (Close et al. 2002). The expression pattern switch of *egr1* in zebrafish embryogenesis is highly similar to the dynamic accessible elements switch in medaka *egr1*. To test whether the enhancer switching logic commonly exists, we first selected the element pairs whose accessibility levels are negatively correlated, and then applied a linear regression to fit gene expression level and accessibility level of each element pair. By using *F*-test cutoff <0.1, we get 9296 element pairs containing 11,766 accessible elements regulating 2428 genes.

The fourth type is early opening. A typical case can be seen in *alcamb*. This gene starts to express at stage 19, whereas elements in the 5′ upstream region and the first intron are open at stage 11, earlier than its expression (Fig. 6E). To consider generally how common accessible elements are open earlier than the expression of corresponding gene, we searched for genes with gradually increased expression and gradually opened accessible elements (Supplemental Methods; Supplemental Fig. S18). For these genes, we compared the differentiation stage when the expression level of the gene, or the accessibility of elements, reaches 50% of their maxima (Fig. 6G). If the accessible elements open together with the increase of expression, we will see genes fall onto the diagonal line. However, strongly skewed patterns were observed: For 3012 genes, 5775 accessible elements became open before the onset of transcription. This suggested that early opening might be a common regulatory pattern in embryogenesis.

### *Trans*-regulatory logic

In addition to the analysis of *cis*-regulatory logic between genes and their nearby DNA elements, we also investigated the *trans*-regulatory logic between genes and TFs. This can be inferred from the TF binding motif enrichment and TF footprints from the ATAC-seq profiles.

Pioneer TFs bind to previously closed chromatin, open the region, and allow other TFs to bind nearby (Zaret and Carroll 2011; Soufi et al. 2012). The protein interaction quantification (PIQ) algorithm calculates a chromatin opening index score on the basis of the frequency of transposase cutting sites surrounding TF footprints. This method has been used to validate and predict new pioneer TFs (Sherwood et al. 2014; Gehrke et al. 2019). Another algorithm, chromVAR (Schep et al. 2017), calculates the motif enrichment of the given TF. We integrated the binding information extracted from these algorithms and the expression information obtained above to predict pioneer TFs in medaka embryogenesis. Those TFs with chromatin opening index score >2.5, chromVAR score >10, and gene expression TPM >5 were defined as pioneer TFs (Supplemental Table S5).

**Figure 7.**
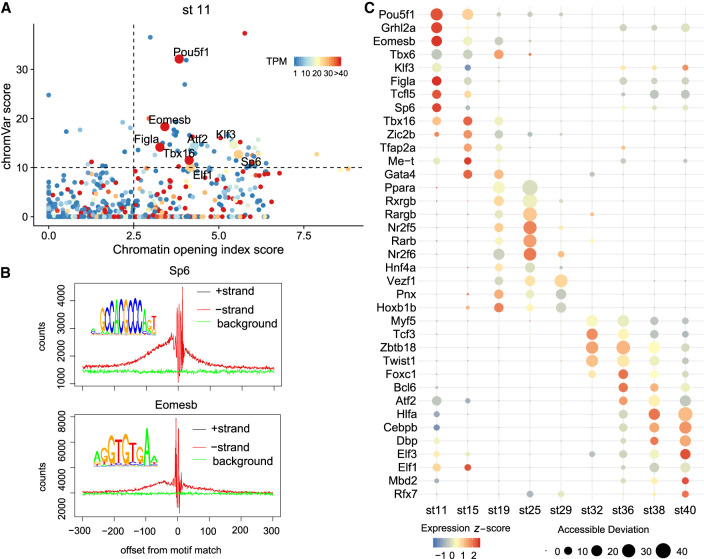
*Trans*-regulatory logic between genes and TFs. (*A*) Pioneer TF prediction at stage 11. Pioneer score is the *x*-axis, chromVAR score is the *y*-axis, and the expression level is represented by the color. Pioneer TFs are those dots with high scores of three measurements, plotted with larger size. (*B*) TF footprint of Sp6 and Eomesb. Within accessible elements, Tn5 insert counts suddenly drop around the given TF binding motif owing to the protection of that TF. (*C*) TF expression and enrichment of its binding motif. The size of the dots was correlated with accessible deviation calculated by chromVAR, indicating the enrichment of TF motifs. The color of the dots reflects the expression level of the given TF.

We found that previously known pioneer TFs were recovered in our data particularly in the relevant stages. For example, in stage 11 (blastula), Pou5f3 (ortholog of human POU5F1), Sp6, and Eomesb were identified as pioneer TFs (Fig. 7A). They also left obvious footprints (the drop of Tn5 insert counts around the TF binding motif) in the accessible elements (Fig. 7B). Genes encoding these TFs are known to play critical roles in early embryogenesis (Pan and Thomson 2007; Lee et al. 2013; Simon et al. 2017). In stage 19 (two-somite) to stage 25 (18/19-somite) when embryonic brain and nerve cord can be recognized, Nr2f6 was recognized as a top candidate (Supplemental Fig. S19). Previous studies showed that Nr2f6 is required in the development of the locus coeruleus, which is the major noradrenergic nucleus of the brain (Warnecke et al. 2005). By using ATAC-seq data from multiple species discussed above, we compared chromatin opening index scores among human, mouse, chicken, zebrafish and medaka and found that these pioneer scores are highly correlated (Supplemental Fig. S20), suggesting a set of common pioneer TFs are used during early embryogenesis in the vertebrate. We believe that many novel pioneer TFs identified in this study will be valuable for further study.

We also investigated the dynamics of TF activities during embryogenesis, integrating both expression data and DNA motif enrichment of TFs with binding matrices data available (Fig. 7C; Supplemental Fig. S21). The result showed that many TFs are expressed at high levels (orange/red color) when their binding motifs are also significantly enriched in the accessible elements (large size) at the same stages, indicating the critical regulatory functions of these genes at the corresponding stages. For example, retinoic acid (RA) signal-related genes *nr2f6*, *rxrgb*, *rarb*, and *pparaa* are enriched in stages 19–32 when medaka organogenesis is happening. It is known that RA signaling plays a central role during vertebrate development, including neuronal differentiation in the hindbrain and spinal cord, eye morphogenesis, differentiation of forebrain basal ganglia, and heart development (Cunningham and Duester 2015).

The above *cis*- and *trans*-regulatory logic analyses showed that integrating gene expression and chromatin accessibility time-series data sets provided a wealth of information on dynamic gene regulation during embryogenesis.

### Medaka omics data portal

To facilitate the use of these data sets, we built a medaka omics data portal containing three web tools (http://tulab.genetics.ac.cn/medaka_omics/). The first one is the genome browser implemented using JBrowse (Supplemental Fig. S22A; Buels et al. 2016). With this tool, users could compare gene models from the Ensembl, RefSeq, PacBio, and IGDB sets and explore all Illumina and ATAC-seq read coverage and alignments at any genomic locus at each time points. We also incorporated multiple published medaka omics data sets (Nakamura et al. 2014; Tena et al. 2014; Ichikawa et al. 2017; Marlétaz et al. 2018; Uesaka et al. 2019). In total, we constructed 100 tracks to present various annotations, read coverage, and alignments in embryonic stage (24) and adult tissue (seven) samples, using different technologies including RNA-seq (42), ATAC-seq (21), and histone modifications (13). Users can use a sophisticated track selector to search for tracks of interest.

The second tool is a gene viewer for functional annotation and quantification data query and visualization (Supplemental Fig. S22B). This web tool was implemented with R Shiny (R Core Team 2020; https://shiny.rstudio.com), with an SQLite database as the backend. The database contains all gene models in the IGDB set, including their sequences and corresponding Ensembl gene ID, name, GO annotation, Interpro protein domain annotation, and TF family, as well as their orthologs in human, mouse, zebrafish and fly. The essential function of this web tool is to query and visualize the quantification data of gene expression. Users can obtain TPM, percentage, or *z*-scores of expression data for any gene of interest and visualize them using heatmaps or line plots.

The third tool is a BLAST server implemented by sequenceserver (Priyam et al. 2019). Users can search the medaka genome and cDNA sets by their sequences. The genome BLAST report has links to the genome browser to visualize the hits on the genome (Supplemental Fig. S22C). The gene viewer also has links to the genome browser so that users can quickly explore all information of gene of interest. This set of tools makes the enormous amount of medaka omics data easily accessible for all researchers and could become a daily and essential data portal for the community.

## Discussion

### Improving genome annotation with minimum ENCODE toolbox

Extraordinary progress has been made in high-throughput sequencing technologies in the past decade. The widespread use of these technologies generated a vast amount of data and, as a consequence, revealed genome-wide molecular mechanisms underlining complex biological processes at an unprecedented rate (Goodwin et al. 2016). For example, to date in the NCBI Gene Expression Omnibus (GEO) database, a genomics data repository, there are 3,203,367 samples that have been profiled by array- or sequencing-based technologies (https://www.ncbi.nlm.nih.gov/geo/summary/?type=taxfull). Among these profiles, human and mouse have been studied intensively, which contribute 54% and 25% of samples, respectively, in the GEO database. These data sets provide a tremendous amount of information regarding the activity and function of human and mouse genomes under various conditions (The ENCODE Project Consortium 2012; Yue et al. 2014). In contrast, there are significantly fewer genomic data sets available for other organisms. At the organism list sorted by sample numbers, the next eight organisms collectively account for 10% of all archived data, with the remaining approximately 4600 organisms contributing 11% (Supplemental Table S6). Therefore, for most organisms, the research resources are very limited. How to maximize the information that could be acquired from limited research resources is a big challenge.

The ENCODE Consortium uses a set of genomic assays as the standard, including DNA binding (ChIP-seq for histones and TFs), DNA accessibility (ATAC-seq, DNase-seq), DNA methylation (WGBS), 3D chromatin structure (ChIA-PET, Hi-C), transcription (bulk RNA-seq, long read RNA-seq, small RNA-seq, etc.), and RNA binding (eCLIP, RNA Bind-N-Seq) (https://www.encodeproject.org/data-standards/). The Consortium has developed protocols and guidelines for these technologies. Nevertheless, it is demanding for any single laboratory to apply all such metrics to their organism of interest.

In this study, we generated three genomic data sets: PacBio long-read RNA-seq for gene model construction, Illumina short-read RNA-seq for transcript abundance quantification, and ATAC-seq for identification of potential regulatory elements. We refer to these three technologies as a minimum ENCODE toolbox.

Technically, they are all simple enough for most molecular biology laboratories. In our experience, the vital technical requirement of RNA-seq is to extract HQ RNA and of ATAC-seq is to purify HQ nuclei. Other than that, there are no significant requirements to prevent acquiring such data sets. Bioinformatics algorithms for analyzing these assays are rather straightforward. Lastly, bespoke antibodies are not required, which is critical for DNA binding assays, whereas it is often not available for many organisms.

On the other hand, the information obtained from these assays is of great value. Take this study as an example; we acquired information as follows:

1. A set of accurate gene models for protein-coding RNAs and lncRNAs. This is the most fundamental information for all other genomic metrics. As discussed above, gene models from many organisms are based on computational prediction, comparative genomics, and short-read RNA-seq, all of which carry inherent limitations. Long-read RNA-seq is the direct measurement of full-length cDNAs and provides a solid foundation for gene and isoform annotation.
2. Quantification of transcript abundance. This is another critical piece of information that indicates the activity of genes. High-throughput short-read RNA-seq has been used for this purpose for years and has become a standard method to measure the expression level of genes on a genome-wide scale.
3. A set of potential *cis*-regulatory elements. Perhaps the most elusive genomic information concerns DNA regulatory elements. Despite many assays used by the ENCODE project dedicated to this task, most require bespoke antibodies and sophisticated experimental techniques. Contrariwise, ATAC-seq reflects DNA accessibility, which is a proxy of DNA regulatory function. Although this assay is unable to distinguish individual TF binding sites, it does locate regions of the genome that likely harbor regulatory elements.
4. Dynamics of gene expression and DNA element accessibility. Profiling such metrics across developmental time informs upon the dynamics of genome activity. Biological events such as isoform switching, enhancer switching, etc., can be discovered from the profiles of genome activity. Critical regulatory genes and DNA elements underlining these events could be identified in further studies.

Given the straightforward experimental and computational protocols involved and the rich information thus obtained, we propose that the minimum ENCODE toolbox (long-read RNA-seq, short-read RNA-seq, and ATAC-seq) could serve as the initial and essential profiling assays for model organisms lagging behind in terms of available genomic information.

### Limitations

Our current annotation and data sets still have some limitations. The major one is the limited depth of long-read RNA-sequencing. PacBio sequencing is still costly compared with high-throughput Illumina sequencing. Therefore, the biological samples profiled in this study were limited, and pooling multiple samples into one sequencing library made the sequencing depth not deep enough. In the end, the PacBio gene models contribute 61% of the final gene set. Some genes or isoforms expressed at a low level or in the restricted domains are likely missed. For example, *vasa* is a germ cell specifically expressed gene (Shinomiya et al. 2000). In medaka embryos, *vasa* transcript presents in each blastomere at the early stages and then restricts to germ cells, which are only a tiny proportion of the whole embryo at late stages. In medaka adults, it is highly expressed in both ovary and testis. In our PacBio sequencing results, this transcript is detected in all libraries except the late-stage library pooled from stages 32, 36, 38, and 40. At these stages, there are only about 60 to 140 germ cells per embryo (Kobayashi et al. 2004). Thus, if a gene is expressed in such a small proportion of the embryo, our current sequencing depth is not able to detect the transcript.

lncRNAs could also be missed as they are likely expressed at lower levels with a time- or space-limited manner. For example, *LDAIR* is a newly identified seasonal regulated lncRNA, which is transcribed from the first intron of *lpin2* but in the opposite direction (Nakayama et al. 2019). Our PacBio set failed to detect this transcript. However, it was recovered from Illumina short reads and showed to be transiently expressed during stages 15–25 (Supplemental Fig. S23). However, all our data sets, including PacBio reads, Illumina reads, and ATAC-seq reads, showed that the Ensembl isoforms of *lpin2* were not accurate, at least for the biological samples surveyed in this study. Our data sets identified two longer isoforms and its highly accessible promoter as well.

## Methods

### Fish

All fish experiments followed the guidelines of the animal care and use committee of the Institute of Genetics and Developmental Biology, Chinese Academy of Sciences. Three medaka strains, Hd-rR, d-rR-Tg (olvas-GFP) (Tanaka et al. 2001), and Qurt (Wada et al. 1998), were used in this study. All three strains were obtained from the Japan NBRP Medaka resource center (https://shigen.nig.ac.jp/medaka/). Fish were maintained in freshwater at 28°C under photo-periodically regulated conditions (14-h light and 10-h dark).

### Sample collection and RNA extraction

Stages of development for embryos were determined by eye based on medaka normal developmental stages (Iwamatsu 2004). About 20-400 embryos were collected per stage depending on the RNA yield per embryo. Samples of different developmental stages were collected from synchronous embryos to ensure that all embryos were at the same developmental stage. All time course samples for RNA-seq surveyed in this study had two replicates. Adult organ samples were dissected using scissors and tweezers, and larval gonads were obtained by microdissection.

RNA was extracted using both TRIzol (Thermo Fisher Scientific 15596-018) and RNeasy Micro Kit (Qiagen 74004) with a modified protocol to ensure the complete lysis and high RNA yield. For details, see Supplemental Methods.

### RNA sequencing

For long-read RNA sequencing, barcoded SMRTBell libraries were sequenced on a PacBio Sequel platform. PacBio raw reads were first processed by the PacBio Iso-Seq pipeline to generate HQ, full-length, transcript isoform sequences.

For short-read RNA sequencing, stranded cDNA libraries of 13 samples were generated using TruSeq stranded mRNA library prep (Illumina 20020594) and sequenced on the Illumina HiSeq X TEN platform (PE150). Quality control was performed using the FastQC package, the FASTX-toolkit, and the RSeQC package. Reads were mapped to the medaka reference genome using HISAT2 (Kim et al. 2015). Quantification of genes and isoforms was performed using StringTie (Pertea et al. 2015, 2016) with the IGDB gene model constructed in this study. For details, see Supplemental Methods.

In this study, all RNA-seq and ATAC-seq libraries were sequenced by Annoroad Gene Technology.

### Gene model construction and analysis

PacBio HQ reads were mapped to the medaka reference genome with Spaln2 (Iwata and Gotoh 2012), and redundant transcripts models were collapsed using the merge mode of StringTie. Short-read coverage of splice junctions observed in the PacBio set was calculated using QoRTs. A different gene model set was compared using gffcompare program (Pertea and Pertea 2020). Finally, we integrated the PacBio set and Ensembl set gene models into one set (IGDB set).

For TF annotation, the protein sequences of IGDB set ORFs were screened using the TF prediction tool of AnimalTFDB 3.0 to obtain the TF family annotation. For lncRNA annotation, we scored the coding potential of sequences with CPAT and CPC software to remove the high coding potential transcripts. Time course profiling data (TPM) were standardized using the *z*-score method. Clustering analysis was performed using “Mfuzz” (R package). GO term enrichment analysis was performed using “clusterProfiler” (R package). For details, see Supplemental Methods.

### ATAC-seq and analysis

ATAC-seq libraries were prepared as previously reported with some modifications. All ATAC-seq samples surveyed in this study had two replicates. All ATAC-seq libraries were sequenced on the Illumina HiSeq X TEN platform.

Reads were trimmed and mapped to the reference genome. Sample replicates were merged for downstream analysis. Peak calling was performed with MACS2 (Zhang et al. 2008). Peaks from all stages were filtered with a stringent cutoff (−log(*q*-value) > 7) and then merged for downstream analysis. Accessible element distribution was calculated according to genomic location.

Medaka conserved regions data among 51 fish genomes were download from Ensembl Compara. Accessible elements that overlapped with constrained regions were defined as conserved. RS score of each conserved accessible element was represented by the maximum RS scores of all overlapped constrained regions.

When calculating the early opening of accessible elements, we compared the Spearman's correlation value between promoter–gene pairs and randomly picked promoter–gene pairs. The corresponding *P*-value was calculated, and only those genes showing a *P*-value ≤ 0.1 (Spearman's correlation coefficient > 0.7) were retained.

TF binding motif enrichment was calculated using chromVAR. We first forged a BSgenome package for *O. latipes*. We used chromVAR with default parameters according to a standard walkthrough. For motif matching, we collected human, zebrafish, and medaka motifs from the CIS-BP database (Weirauch et al. 2014; Lambert et al. 2019). For all motifs, frequencies were first normalized by the LICORS package with tolerance level 10^−6^. Frequencies were then renormalized to sum to one, before a 0.008 pseudo count was added.

The chromatin opening index score was calculated using PIQ. The source code for PIQ was modified to run with the *O. latipes* genome. To get a panoramic chromatin opening index score, PIQ was run according to default parameters using default JASPAR motifs (Fornes et al. 2020) and CIS-BP motifs as previously described. For details, see Supplemental Methods.

## Data access

The long-read RNA-seq data generated in this study have been submitted to the NCBI BioProject database (https://www.ncbi.nlm.nih.gov/bioproject/) under accession number PRJNA561263. The short-read RNA-seq data (including gene models in GFF, cDNA sequences, lists of TF/lncRNA IDs, and gene expression table) generated in this study have been submitted to the NCBI Gene Expression Omnibus (GEO; https://www.ncbi.nlm.nih.gov/geo/) (Fornes et al. 2020) under accession number GSE136018. The ATAC-seq data (including peak annotation in BED format, read coverage in bigWig format) generated in this study have been submitted to the NCBI GEO under accession number GSE136027. The data visualization tools can be accessed on our data portal webpage (http://tulab.genetics.ac.cn/medaka_omics/).

## Competing interest statement

The authors declare no competing interests.

## Supplementary Material

Supplemental Material
