# Supplementary material for: Dynamic transcriptional and chromatin accessibility landscape of medaka embryogenesis
Source: Genome Res. 2020 Jun;30(6):924–37. doi: 10.1101/gr.258871.119 (PMC7370878; doi:10.1101/gr.258871.119)
Supplement: Supplemental Material [file supp_gr.258871.119_Supplemental_Fig_S1.pdf]

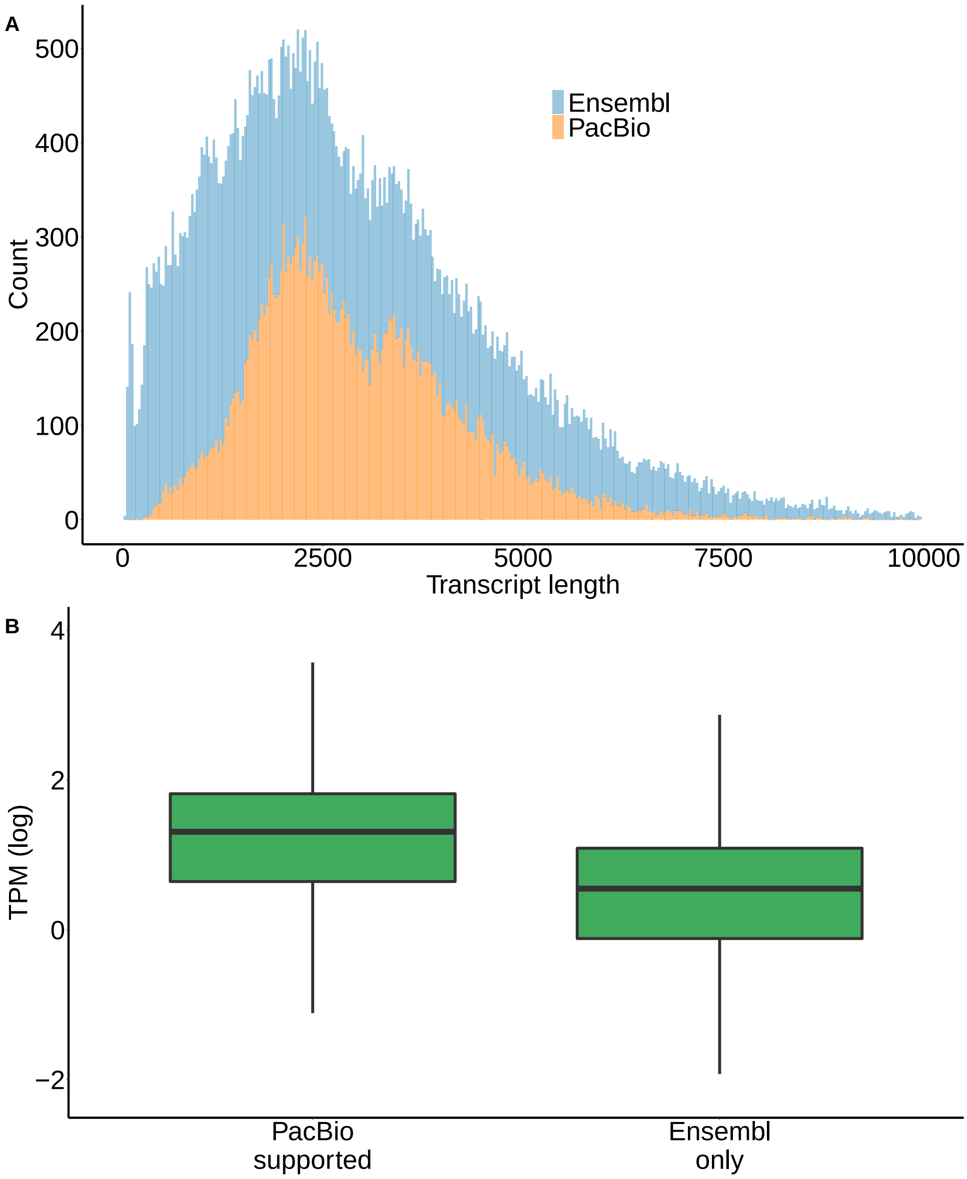

Supplementary Figures 1: (A) Length distributions of Ensembl transcripts and overlapping PacBio transcripts. About 65% of Ensembl transcripts overlap with PacBio transcripts. (B) Transcript abundance of PacBio-supported and Ensembl-only models. PacBio-supported models are gene models in Ensembl model set that have the corresponding PacBio set models. Ensembl-only models have no corresponding PacBio models. The gene expression level of the latter was much lower than that of the former.
