# Supplementary material for: Dynamic transcriptional and chromatin accessibility landscape of medaka embryogenesis
Source: Genome Res. 2020 Jun;30(6):924–37. doi: 10.1101/gr.258871.119 (PMC7370878; doi:10.1101/gr.258871.119)
Supplement: Supplemental Material [file supp_gr.258871.119_Supplemental_Fig_S3.pdf]

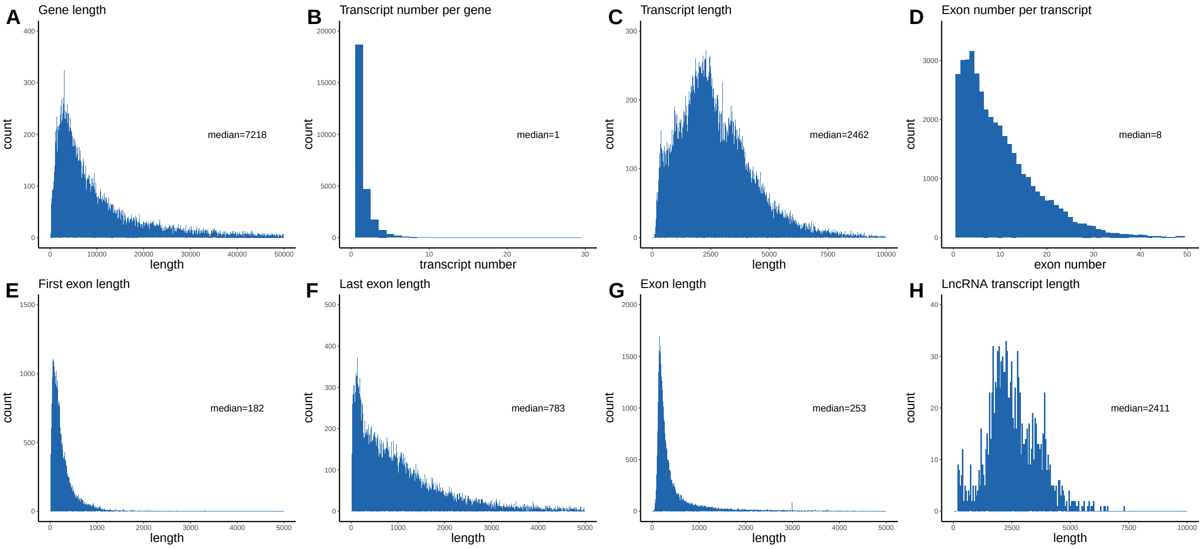

Supplementary Figures 3: Characteristics of IGDB models. (A-H) Distributions of gene length, number of transcripts per gene, transcript length, number of exons per transcript, first exon length, last exon length, exon length, lncRNA transcript length.
