# Supplementary figures and images for: Dynamic transcriptional and chromatin accessibility landscape of medaka embryogenesis
Source: Genome Res. 2020 Jun;30(6):924–37. doi: 10.1101/gr.258871.119 (PMC7370878; doi:10.1101/gr.258871.119)

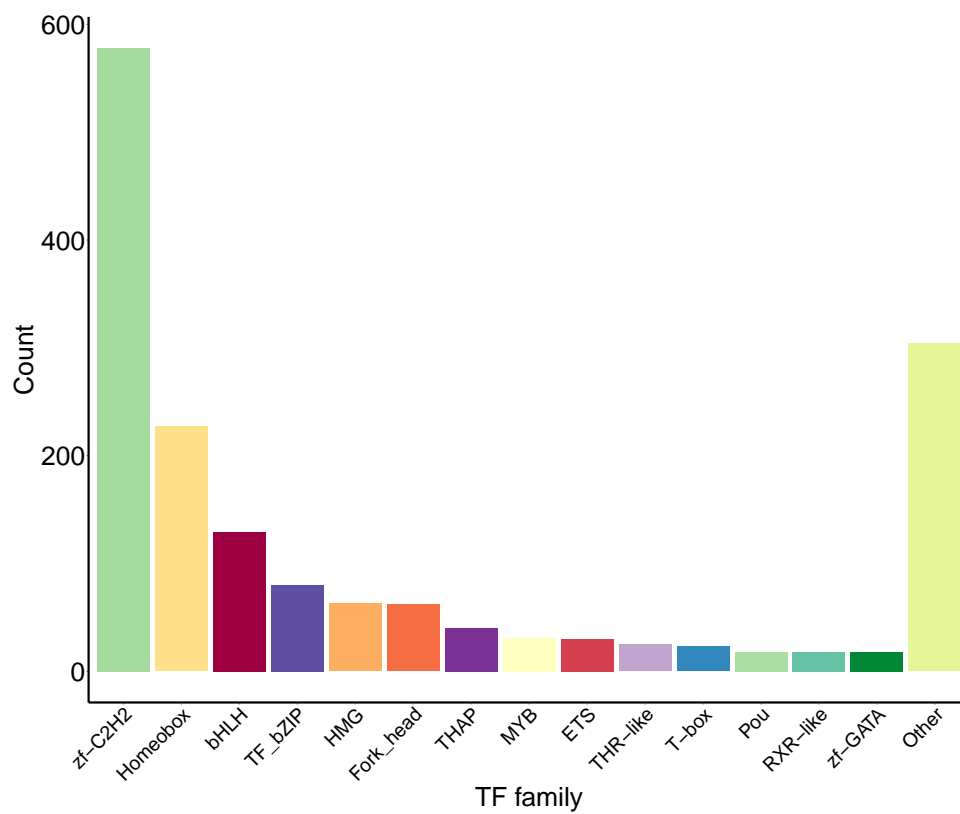

**Supplementary Figures 4:** Transcription factors families and gene number in each family.

Supplement: Supplemental Material [file supp_gr.258871.119_Supplemental_Fig_S4.pdf]

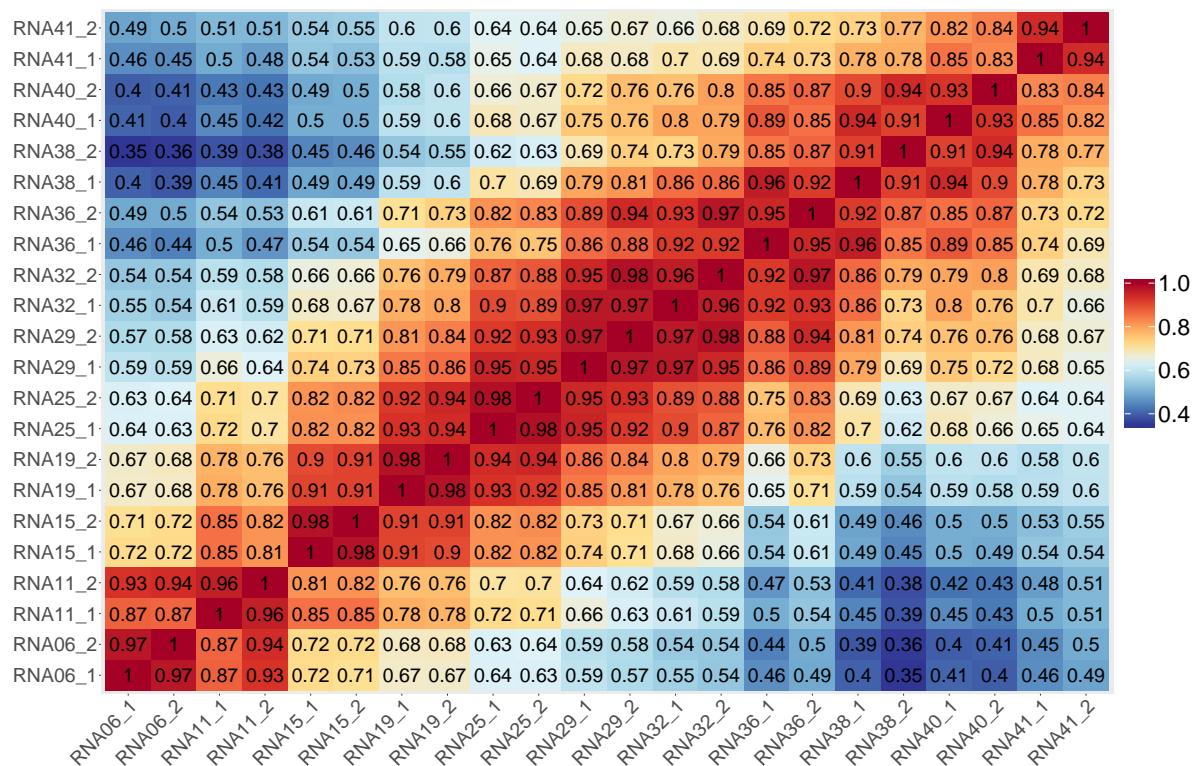

**Supplementary Figures 7:** Spearman's correlations among 11 RNA-seq samples and two replicates.

Supplement: Supplemental Material [file supp_gr.258871.119_Supplemental_Fig_S7.pdf]
