# Supplementary material for: Dynamic transcriptional and chromatin accessibility landscape of medaka embryogenesis
Source: Genome Res. 2020 Jun;30(6):924–37. doi: 10.1101/gr.258871.119 (PMC7370878; doi:10.1101/gr.258871.119)
Supplement: Supplemental Material [file supp_gr.258871.119_Supplemental_Fig_S9.pdf]

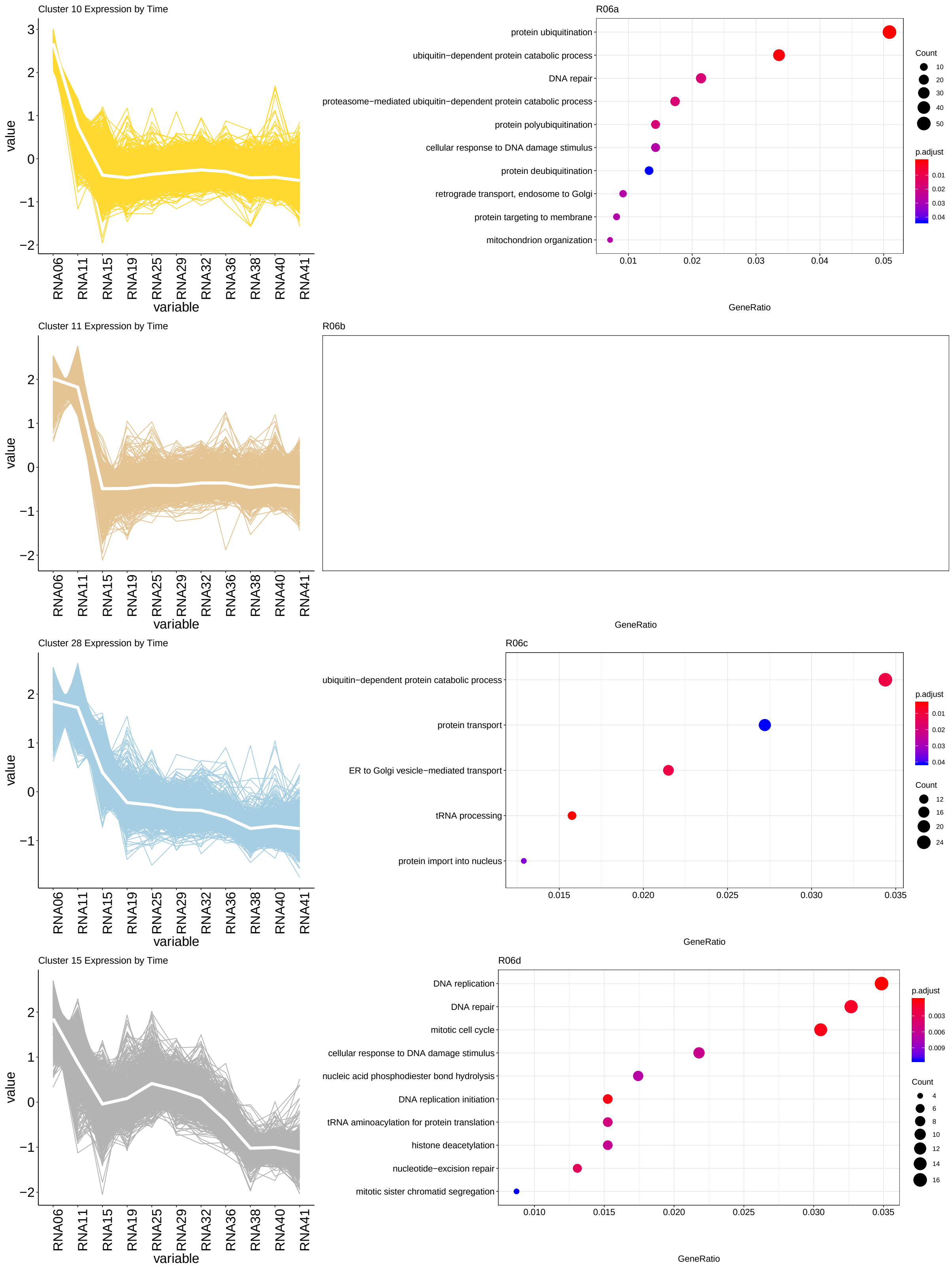
Cluster 10 Expression by Time
value
variable
RNA06
RNA11
RNA15
RNA19
RNA25
RNA29
RNA32
RNA36
RNA38
RNA40
RNA41
R06a
protein ubiquitination
ubiquitin−dependent protein catabolic process
DNA repair
proteasome−mediated ubiquitin−dependent protein catabolic process
protein polyubiquitination
cellular response to DNA damage stimulus
protein deubiquitination
retrograde transport, endosome to Golgi
protein targeting to membrane
mitochondrion organization
GeneRatio
Count
p.adjust
Cluster 11 Expression by Time
R06b
GeneRatio
Cluster 28 Expression by Time
R06c
ubiquitin−dependent protein catabolic process
protein transport
ER to Golgi vesicle−mediated transport
tRNA processing
protein import into nucleus
GeneRatio
p.adjust
Count
Cluster 15 Expression by Time
R06d
DNA replication
DNA repair
mitotic cell cycle
cellular response to DNA damage stimulus
nucleic acid phosphodiester bond hydrolysis
DNA replication initiation
tRNA aminoacylation for protein translation
histone deacetylation
nucleotide−excision repair
mitotic sister chromatid segregation
GeneRatio
p.adjust
Count

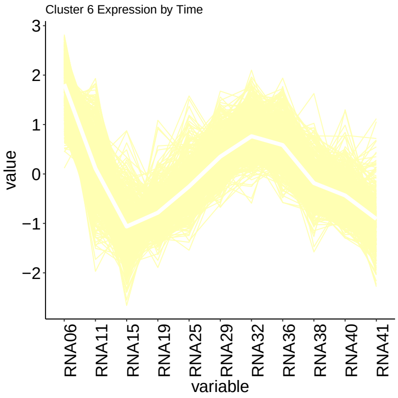
Cluster 6 Expression by Time
value
variable
RNA06 RNA11 RNA15 RNA19 RNA25 RNA29 RNA32 RNA36 RNA38 RNA40 RNA41

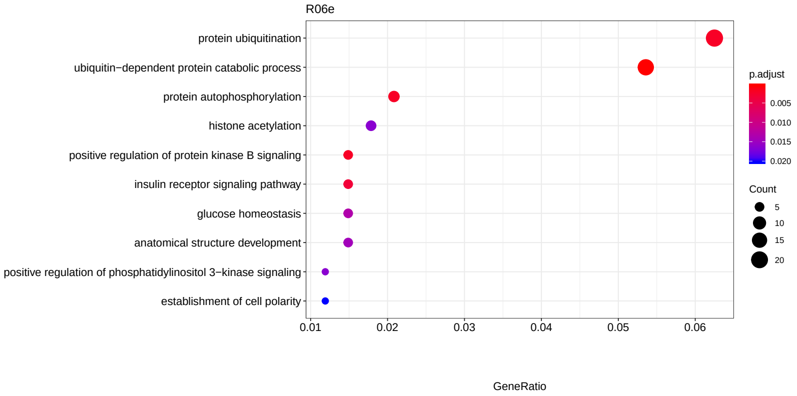
R06e
protein ubiquitination
ubiquitin−dependent protein catabolic process
protein autophosphorylation
histone acetylation
positive regulation of protein kinase B signaling
insulin receptor signaling pathway
glucose homeostasis
anatomical structure development
positive regulation of phosphatidylinositol 3−kinase signaling
establishment of cell polarity
p.adjust
Count
GeneRatio

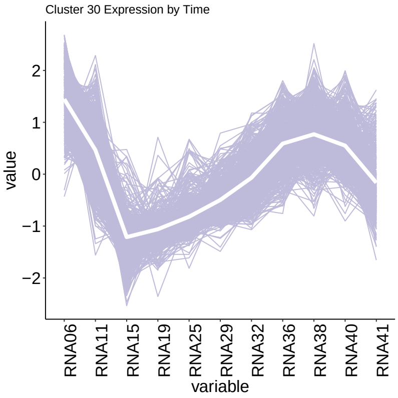
Cluster 30 Expression by Time
value
variable
RNA06 RNA11 RNA15 RNA19 RNA25 RNA29 RNA32 RNA36 RNA38 RNA40 RNA41

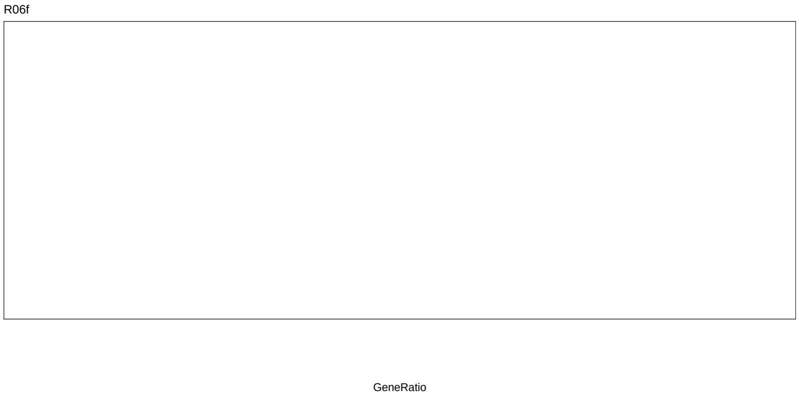
R06f
GeneRatio

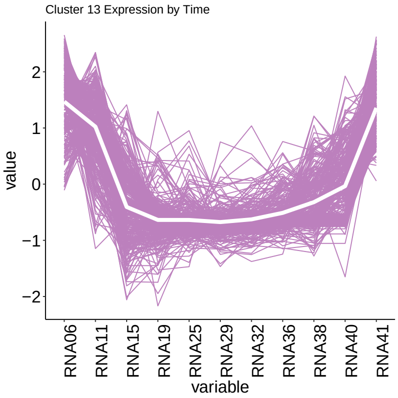
Cluster 13 Expression by Time
value
variable
RNA06 RNA11 RNA15 RNA19 RNA25 RNA29 RNA32 RNA36 RNA38 RNA40 RNA41

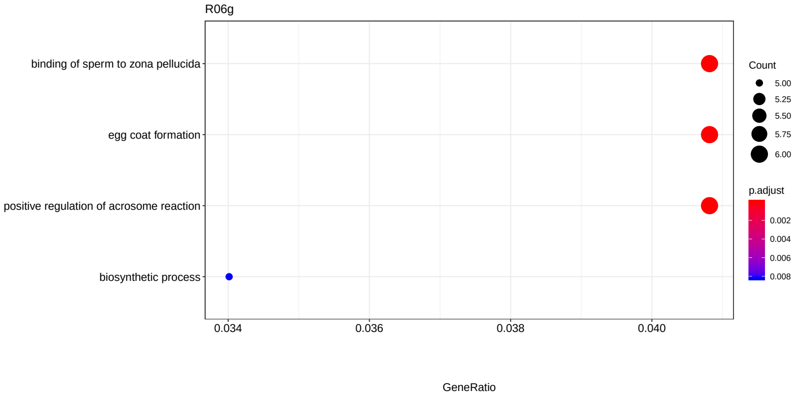
R06g
binding of sperm to zona pellucida
egg coat formation
positive regulation of acrosome reaction
biosynthetic process
Count
p.adjust
GeneRatio

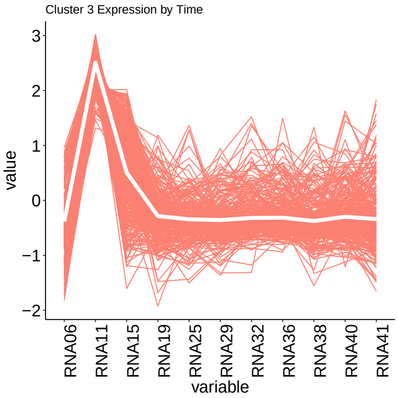
Cluster 3 Expression by Time
value
variable
RNA06 RNA11 RNA15 RNA19 RNA25 RNA29 RNA32 RNA36 RNA38 RNA40 RNA41

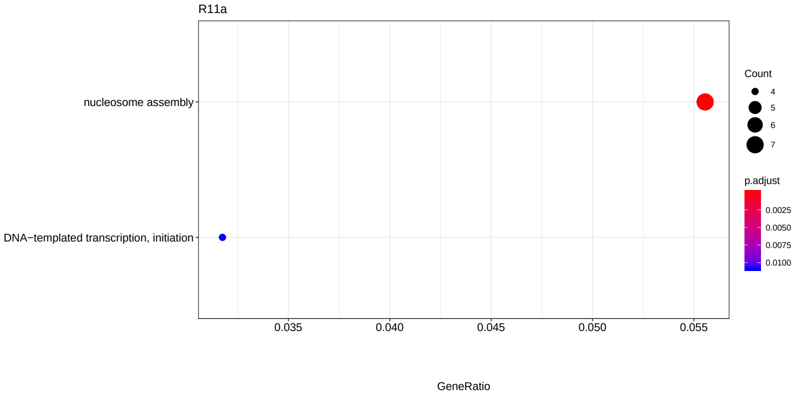
R11a
nucleosome assembly
DNA−templated transcription, initiation
Count
p.adjust
GeneRatio

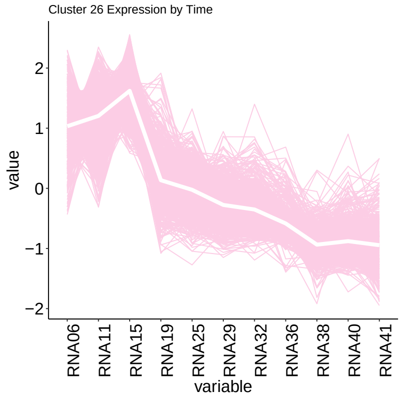
Cluster 26 Expression by Time
value
variable
RNA06
RNA11
RNA15
RNA19
RNA25
RNA29
RNA32
RNA36
RNA38
RNA40
RNA41

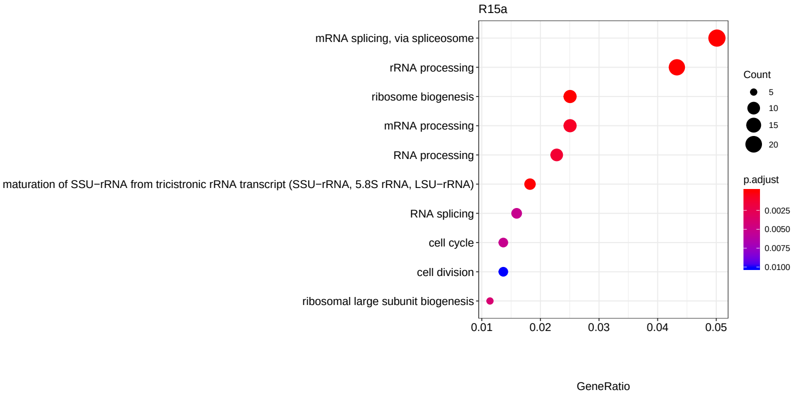
R15a
mRNA splicing, via spliceosome
rRNA processing
ribosome biogenesis
mRNA processing
RNA processing
maturation of SSU−rRNA from tricistronic rRNA transcript (SSU−rRNA, 5.8S rRNA, LSU−rRNA)
RNA splicing
cell cycle
cell division
ribosomal large subunit biogenesis
Count
p.adjust
GeneRatio

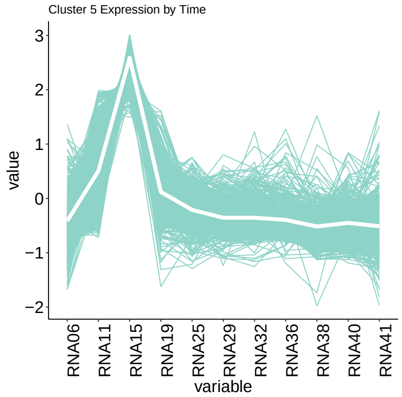
Cluster 5 Expression by Time
value
variable
RNA06
RNA11
RNA15
RNA19
RNA25
RNA29
RNA32
RNA36
RNA38
RNA40
RNA41

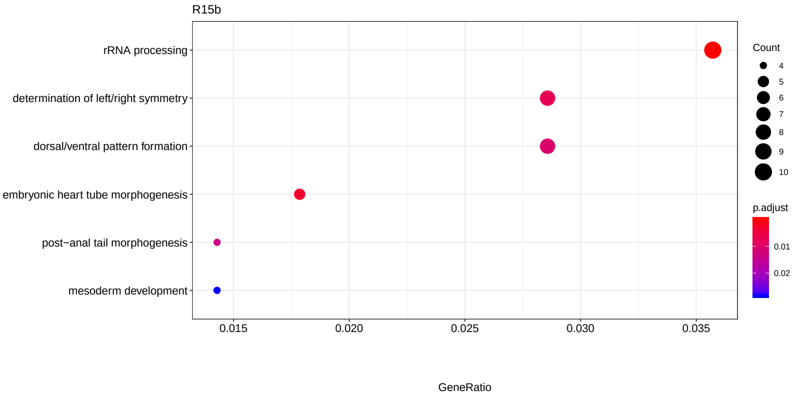
R15b
rRNA processing
determination of left/right symmetry
dorsal/ventral pattern formation
embryonic heart tube morphogenesis
post−anal tail morphogenesis
mesoderm development
Count
p.adjust
GeneRatio

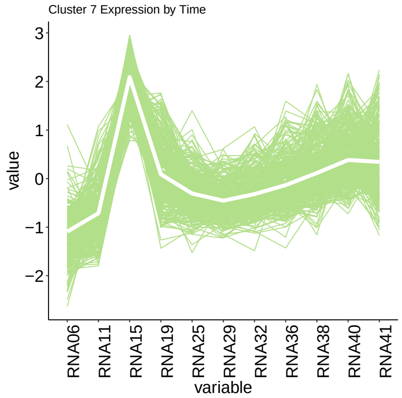
Cluster 7 Expression by Time
value
variable
RNA06
RNA11
RNA15
RNA19
RNA25
RNA29
RNA32
RNA36
RNA38
RNA40
RNA41

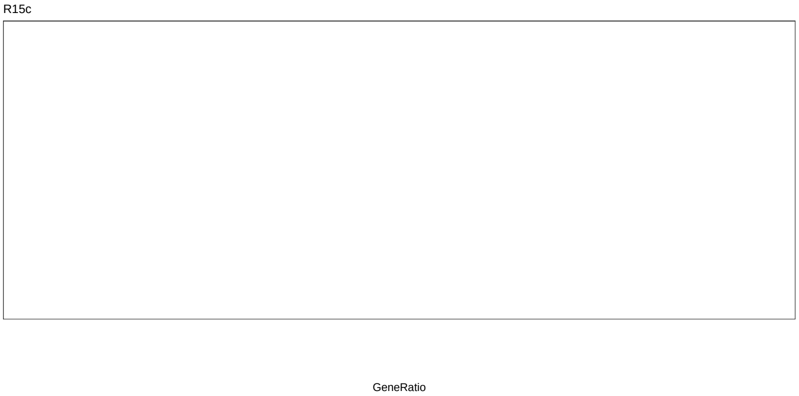
R15c
GeneRatio

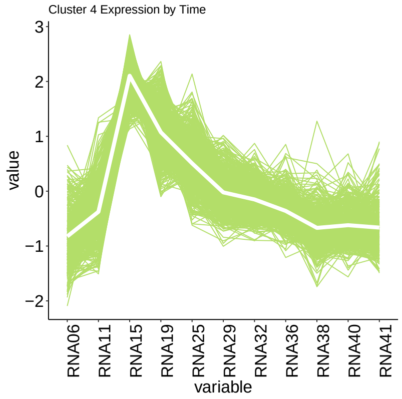
Cluster 4 Expression by Time
value
variable
RNA06
RNA11
RNA15
RNA19
RNA25
RNA29
RNA32
RNA36
RNA38
RNA40
RNA41

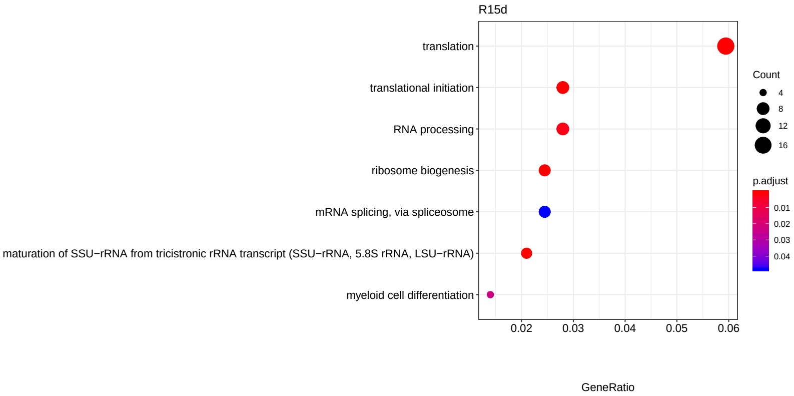
R15d
translation
translational initiation
RNA processing
ribosome biogenesis
mRNA splicing, via spliceosome
maturation of SSU−rRNA from tricistronic rRNA transcript (SSU−rRNA, 5.8S rRNA, LSU−rRNA)
myeloid cell differentiation
Count
p.adjust
GeneRatio

Cluster 8 Expression by Time

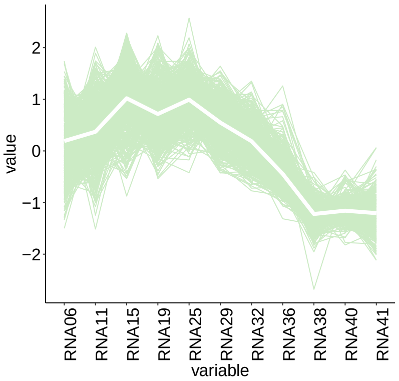

R15e

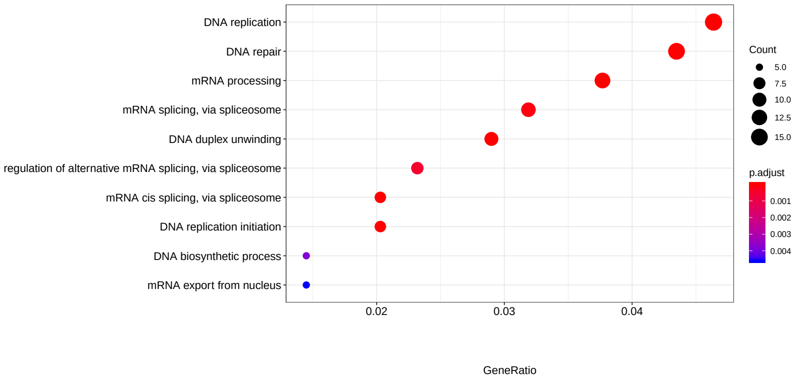

Cluster 21 Expression by Time

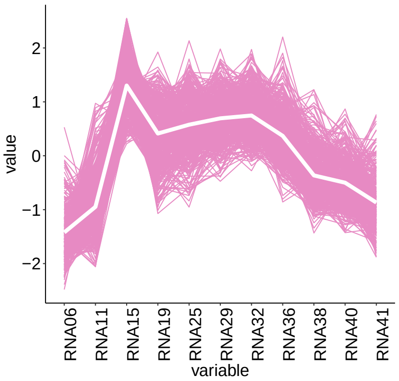

R15f

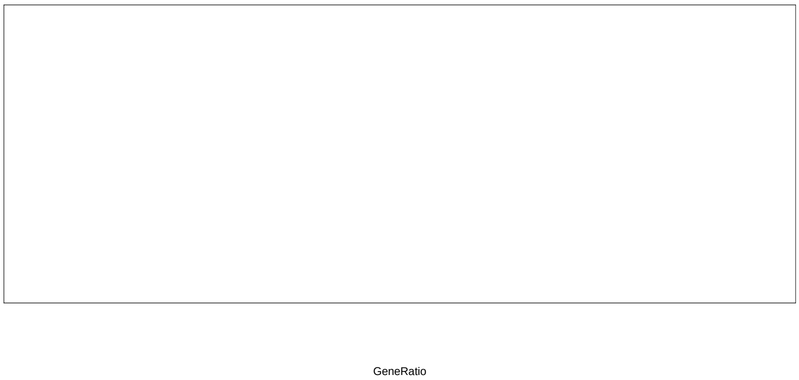

Cluster 12 Expression by Time

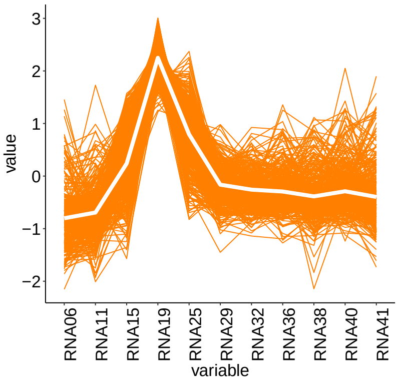

R19a

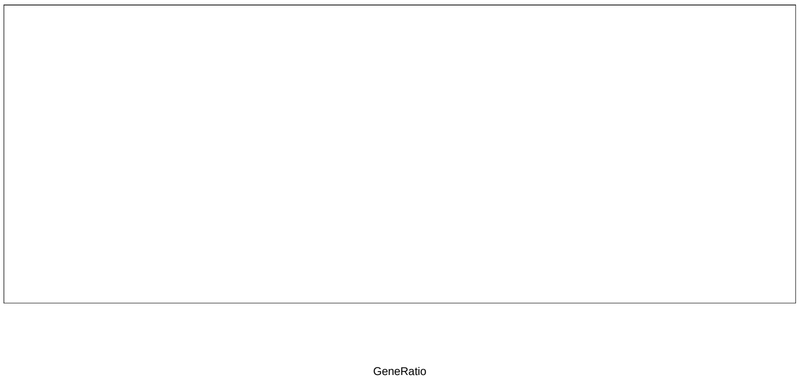

Cluster 17 Expression by Time

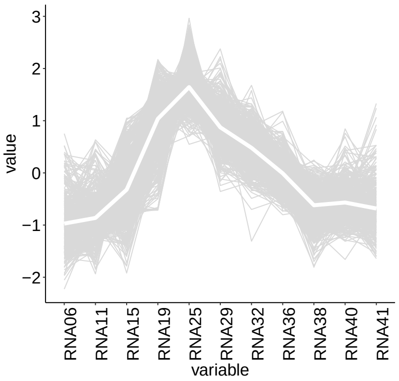

R25a

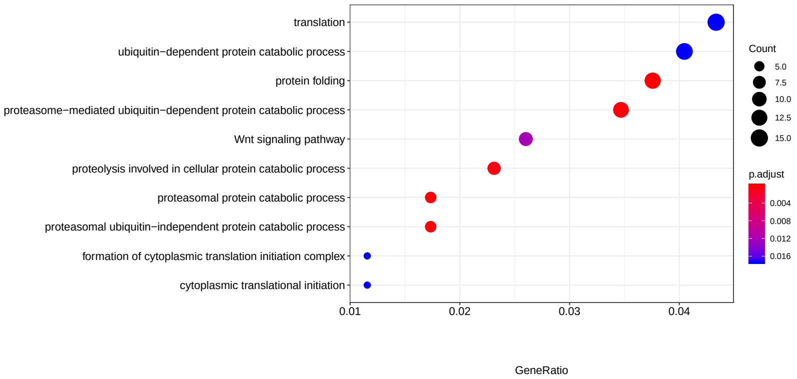

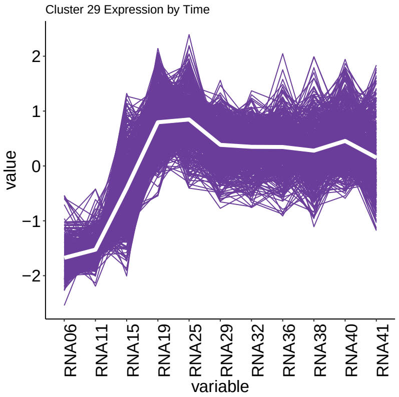
Cluster 29 Expression by Time
value
variable
RNA06 RNA11 RNA15 RNA19 RNA25 RNA29 RNA32 RNA36 RNA38 RNA40 RNA41
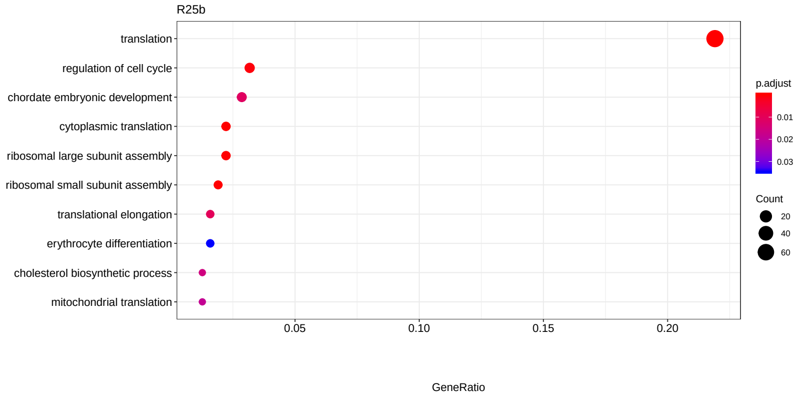
R25b
translation
regulation of cell cycle
chordate embryonic development
cytoplasmic translation
ribosomal large subunit assembly
ribosomal small subunit assembly
translational elongation
erythrocyte differentiation
cholesterol biosynthetic process
mitochondrial translation
GeneRatio
p.adjust
Count
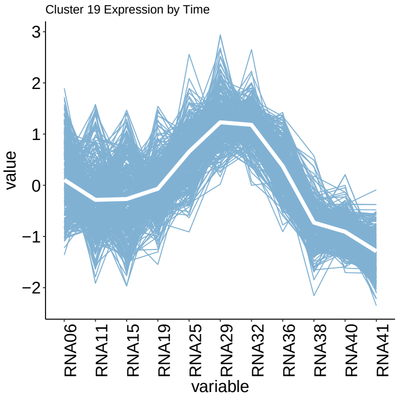
Cluster 19 Expression by Time
value
variable
RNA06 RNA11 RNA15 RNA19 RNA25 RNA29 RNA32 RNA36 RNA38 RNA40 RNA41
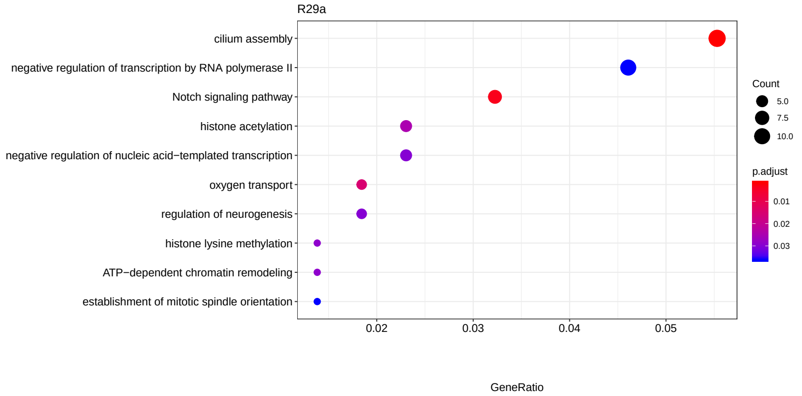
R29a
cilium assembly
negative regulation of transcription by RNA polymerase II
Notch signaling pathway
histone acetylation
negative regulation of nucleic acid−templated transcription
oxygen transport
regulation of neurogenesis
histone lysine methylation
ATP−dependent chromatin remodeling
establishment of mitotic spindle orientation
GeneRatio
Count
p.adjust
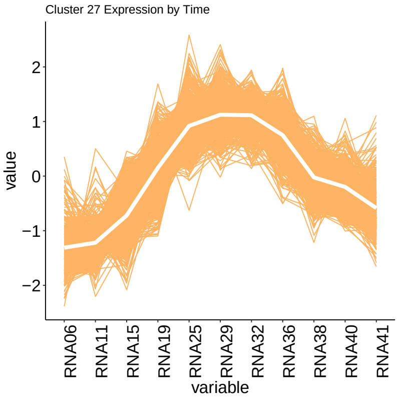
Cluster 27 Expression by Time
value
variable
RNA06 RNA11 RNA15 RNA19 RNA25 RNA29 RNA32 RNA36 RNA38 RNA40 RNA41
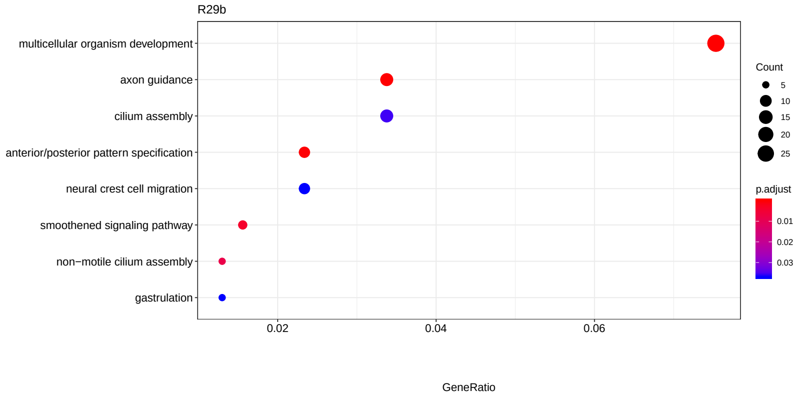
R29b
multicellular organism development
axon guidance
cilium assembly
anterior/posterior pattern specification
neural crest cell migration
smoothened signaling pathway
non−motile cilium assembly
gastrulation
GeneRatio
Count
p.adjust
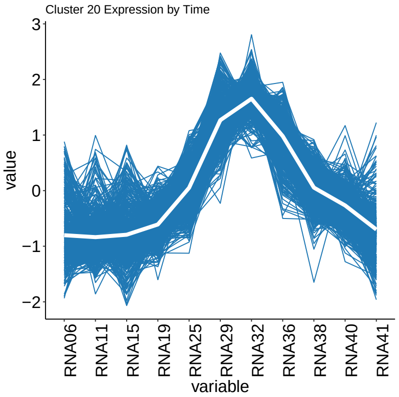
Cluster 20 Expression by Time
value
variable
RNA06 RNA11 RNA15 RNA19 RNA25 RNA29 RNA32 RNA36 RNA38 RNA40 RNA41
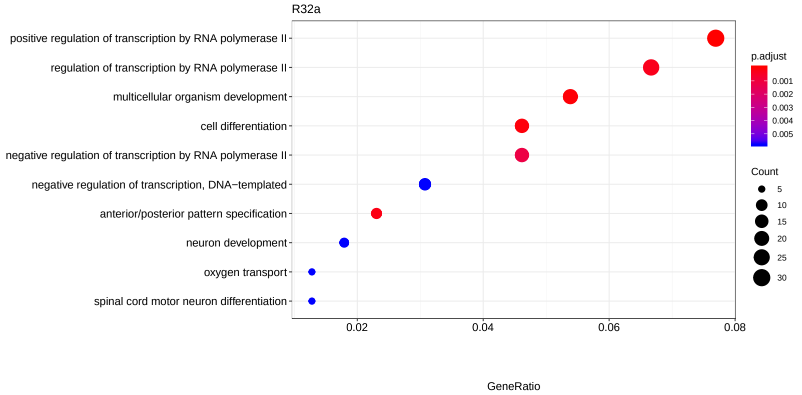
R32a
positive regulation of transcription by RNA polymerase II
regulation of transcription by RNA polymerase II
multicellular organism development
cell differentiation
negative regulation of transcription by RNA polymerase II
negative regulation of transcription, DNA−templated
anterior/posterior pattern specification
neuron development
oxygen transport
spinal cord motor neuron differentiation
GeneRatio
p.adjust
Count

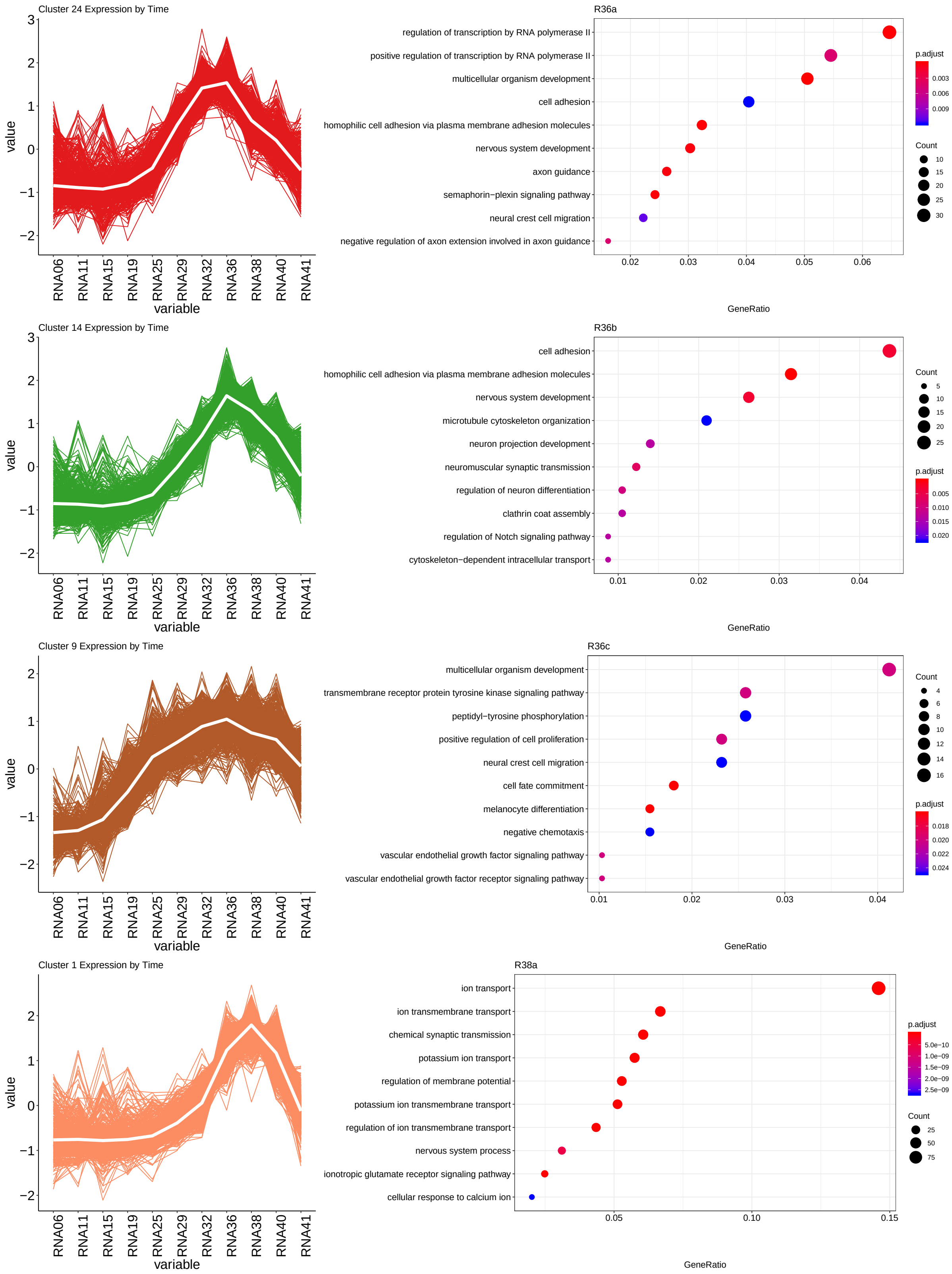

Cluster 24 Expression by Time
value
variable
RNA06 RNA11 RNA15 RNA19 RNA25 RNA29 RNA32 RNA36 RNA38 RNA40 RNA41
R36a
regulation of transcription by RNA polymerase II
positive regulation of transcription by RNA polymerase II
multicellular organism development
cell adhesion
homophilic cell adhesion via plasma membrane adhesion molecules
nervous system development
axon guidance
semaphorin−plexin signaling pathway
neural crest cell migration
negative regulation of axon extension involved in axon guidance
GeneRatio
p.adjust
Count
Cluster 14 Expression by Time
value
variable
R36b
cell adhesion
homophilic cell adhesion via plasma membrane adhesion molecules
nervous system development
microtubule cytoskeleton organization
neuron projection development
neuromuscular synaptic transmission
regulation of neuron differentiation
clathrin coat assembly
regulation of Notch signaling pathway
cytoskeleton−dependent intracellular transport
GeneRatio
Count
p.adjust
Cluster 9 Expression by Time
value
variable
R36c
multicellular organism development
transmembrane receptor protein tyrosine kinase signaling pathway
peptidyl−tyrosine phosphorylation
positive regulation of cell proliferation
neural crest cell migration
cell fate commitment
melanocyte differentiation
negative chemotaxis
vascular endothelial growth factor signaling pathway
vascular endothelial growth factor receptor signaling pathway
GeneRatio
Count
p.adjust
Cluster 1 Expression by Time
value
variable
R38a
ion transport
ion transmembrane transport
chemical synaptic transmission
potassium ion transport
regulation of membrane potential
potassium ion transmembrane transport
regulation of ion transmembrane transport
nervous system process
ionotropic glutamate receptor signaling pathway
cellular response to calcium ion
GeneRatio
p.adjust
Count

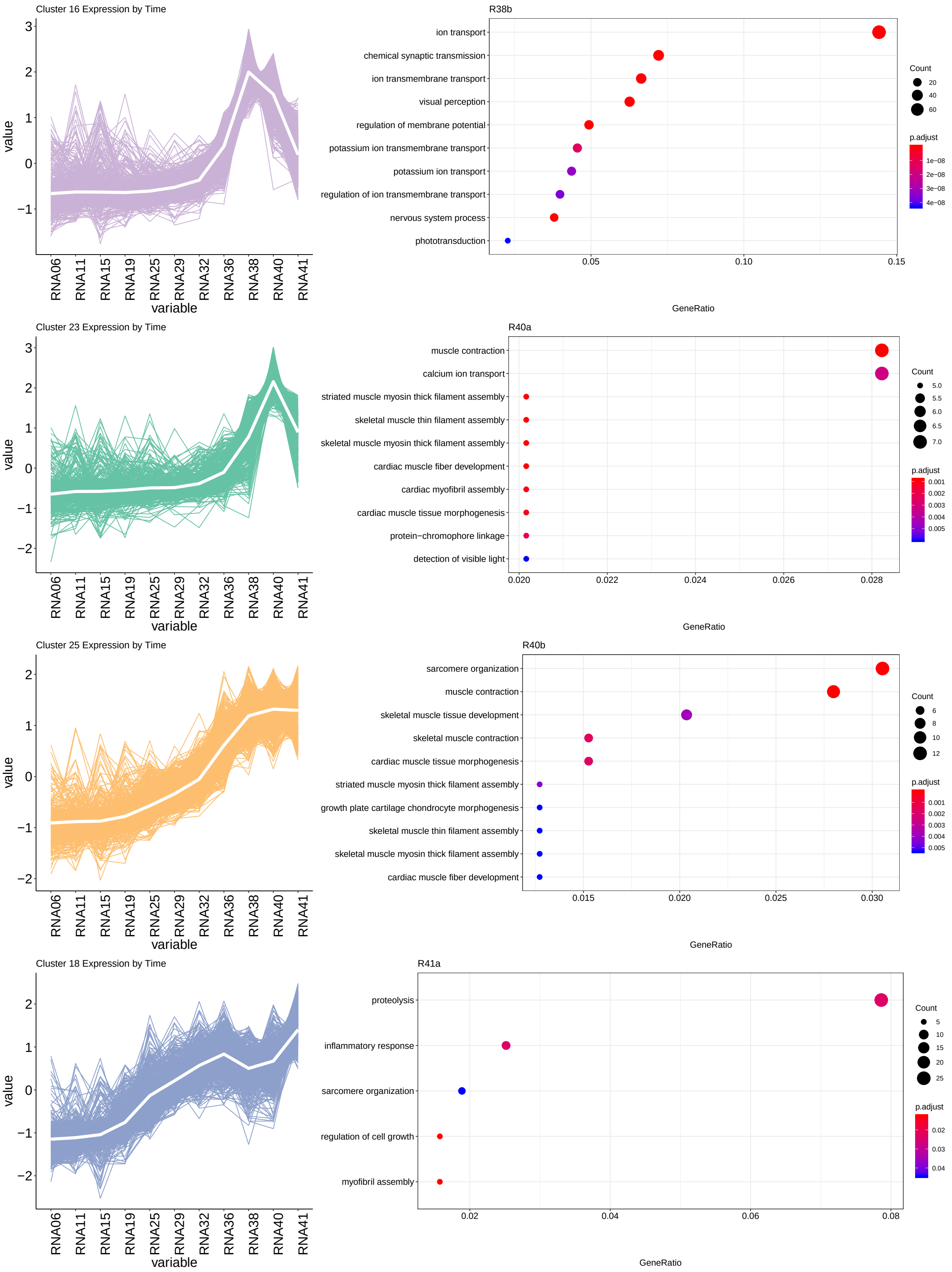
Cluster 16 Expression by Time
value
variable
RNA06 RNA11 RNA15 RNA19 RNA25 RNA29 RNA32 RNA36 RNA38 RNA40 RNA41
R38b
ion transport
chemical synaptic transmission
ion transmembrane transport
visual perception
regulation of membrane potential
potassium ion transmembrane transport
potassium ion transport
regulation of ion transmembrane transport
nervous system process
phototransduction
GeneRatio
Count
p.adjust
Cluster 23 Expression by Time
R40a
muscle contraction
calcium ion transport
striated muscle myosin thick filament assembly
skeletal muscle thin filament assembly
skeletal muscle myosin thick filament assembly
cardiac muscle fiber development
cardiac myofibril assembly
cardiac muscle tissue morphogenesis
protein−chromophore linkage
detection of visible light
Cluster 25 Expression by Time
R40b
sarcomere organization
muscle contraction
skeletal muscle tissue development
skeletal muscle contraction
cardiac muscle tissue morphogenesis
striated muscle myosin thick filament assembly
growth plate cartilage chondrocyte morphogenesis
skeletal muscle thin filament assembly
skeletal muscle myosin thick filament assembly
cardiac muscle fiber development
Cluster 18 Expression by Time
R41a
proteolysis
inflammatory response
sarcomere organization
regulation of cell growth
myofibril assembly

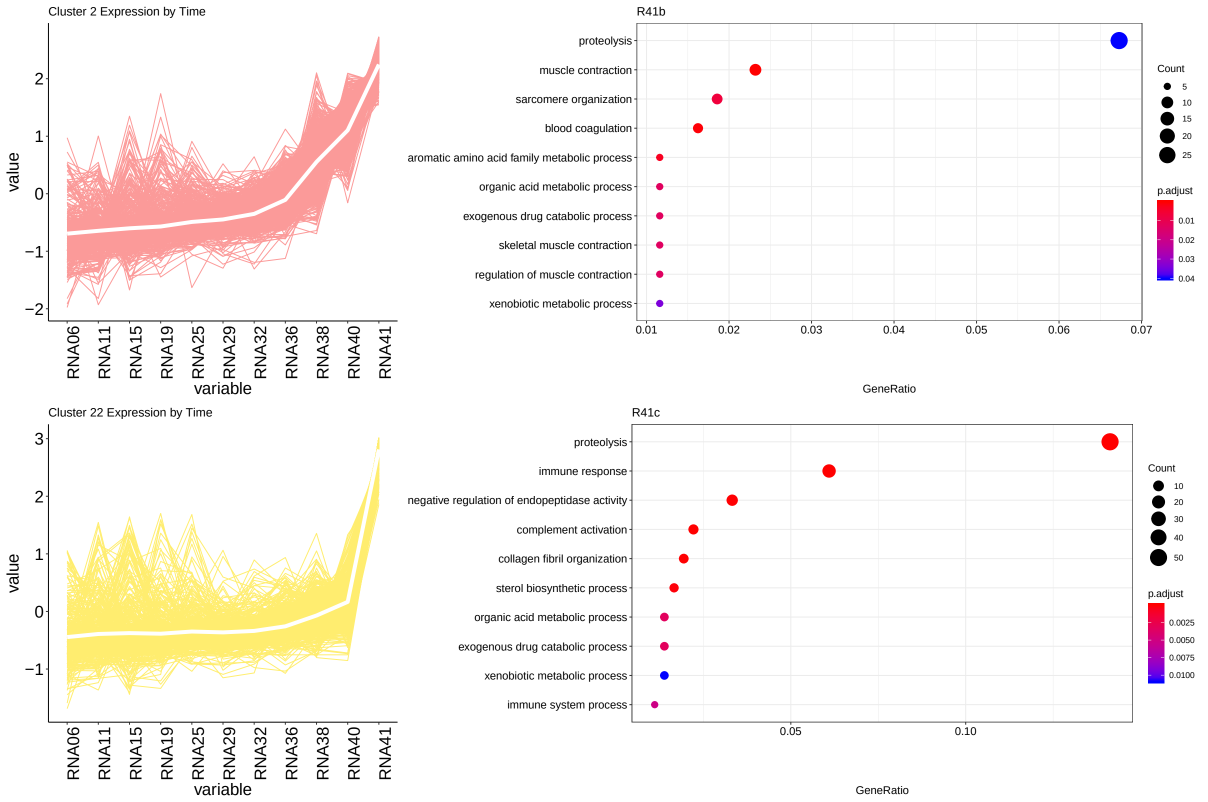

Supplementary Figures 9: Time-series expression profiles and corresponding GO term enrichment of all 30 clusters.
