# Supplementary material for: Dynamic transcriptional and chromatin accessibility landscape of medaka embryogenesis
Source: Genome Res. 2020 Jun;30(6):924–37. doi: 10.1101/gr.258871.119 (PMC7370878; doi:10.1101/gr.258871.119)
Supplement: Supplemental Material [file supp_gr.258871.119_Supplemental_Fig_S11.pdf]

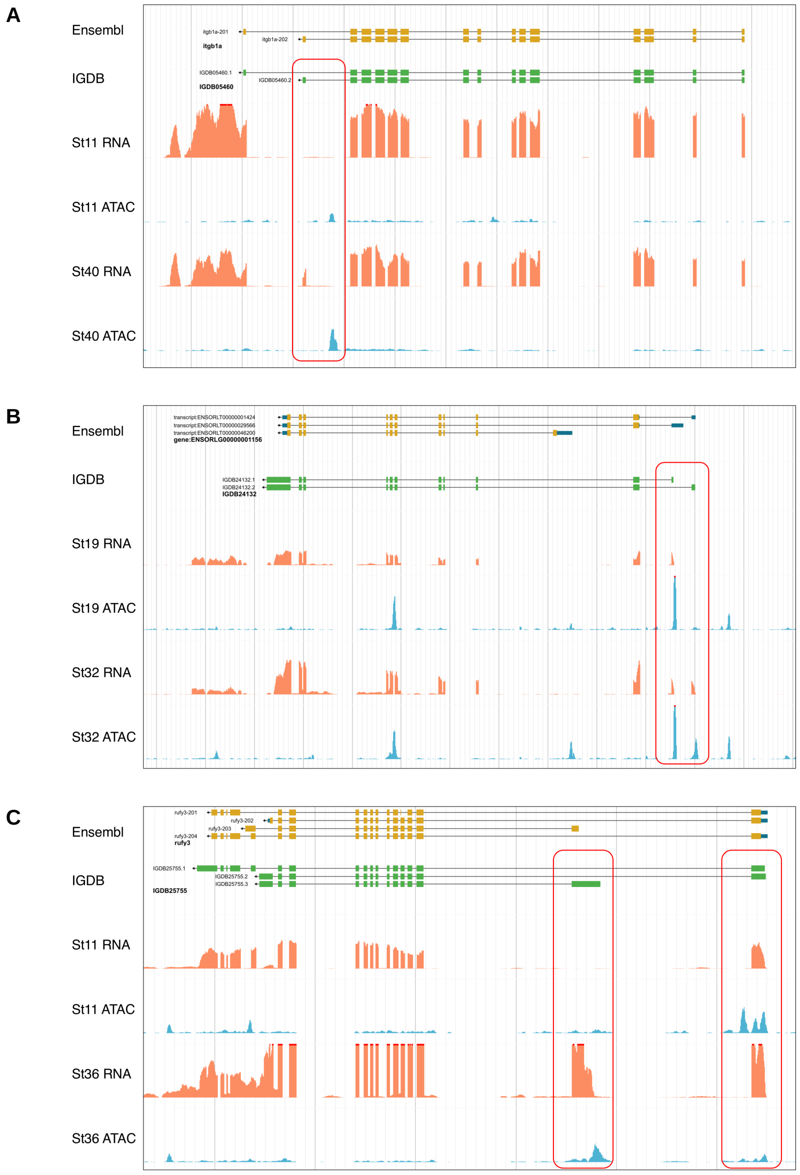

Supplementary Figures 11: Isoform switching and accessible elements switching. (A-C) Isoform switching events of gene IGDB05460, IGDB24132, and IGDB25755, and the corresponding ATAC-seq accessible elements switch in the promoter regions of different isoforms.
