# Supplementary material for: Dynamic transcriptional and chromatin accessibility landscape of medaka embryogenesis
Source: Genome Res. 2020 Jun;30(6):924–37. doi: 10.1101/gr.258871.119 (PMC7370878; doi:10.1101/gr.258871.119)
Supplement: Supplemental Material [file supp_gr.258871.119_Supplemental_Fig_S12.pdf]

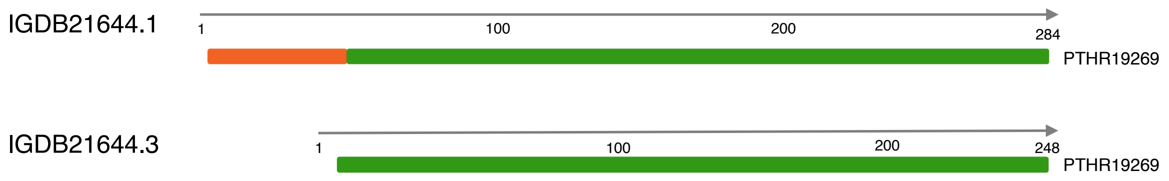


```
IGDB21644.1 MDAIKKKMQMLKLDKENALDRAEQAESDKKASEDRSKQLEDDLVALQKKLKATEDELDKY 60
IGDB21644.3 ------------------------------------MAGGSSLEAVKKKIKSLQEQADAA 24
                                                    ..* *::**:*: ::: *  

IGDB21644.1 SEALKDAQEKLELAEKKATDAEGDVASLNRRIQLVEEELDRAQERLATALTKLEEAEKAA 120
IGDB21644.3 EERAAQLQRDLNQERSAREAAEGDVASLNRRIQLVEEELDRAQERLATALTKLEEAEKAA 84
            .*   : *..*:  ..    ****************************************

IGDB21644.1 DESERGMKVIENRAMKDEEKMELQEIQLKEAKHIAEEADRKYEEVARKLVIIESDLERTE 180
IGDB21644.3 DESERGMKVIENRAMKDEEKMELQEIQLKEAKHIAEEADRKYEEVARKLVIIESDLERTE 144
            ************************************************************

IGDB21644.1 ERAELSEGKCSELEEELKTVQNNLKSLEAQAEKYSQKEDKYEEEIKVLTDKLKEAETRAE 240
IGDB21644.3 ERAELSEGKCSELEEELKTVQNNLKSLEAQAEKYSQKEDKYEEEIKVLTDKLKEAETRAE 204
            ************************************************************

IGDB21644.1 FAERSVAKLEKTIDDLEDELYSQKLKYKAISEELDHALNDMTSI 284
IGDB21644.3 FAERSVAKLEKTIDDLEDELYSQKLKYKAISEELDHALNDMTSM 248
            *******************************************:
```

Supplementary Figures 12: Domain structure of proteins encoded by *tpma* isoforms. The shorter isoform (IGDB21644.3) has one tropomyosin domain (green), while the longer isoform (IGDB21644.1) has an extra partial tropomyosin like domain (orange).
