# Supplementary material for: Dynamic transcriptional and chromatin accessibility landscape of medaka embryogenesis
Source: Genome Res. 2020 Jun;30(6):924–37. doi: 10.1101/gr.258871.119 (PMC7370878; doi:10.1101/gr.258871.119)
Supplement: Supplemental Material [file supp_gr.258871.119_Supplemental_Fig_S13.pdf]

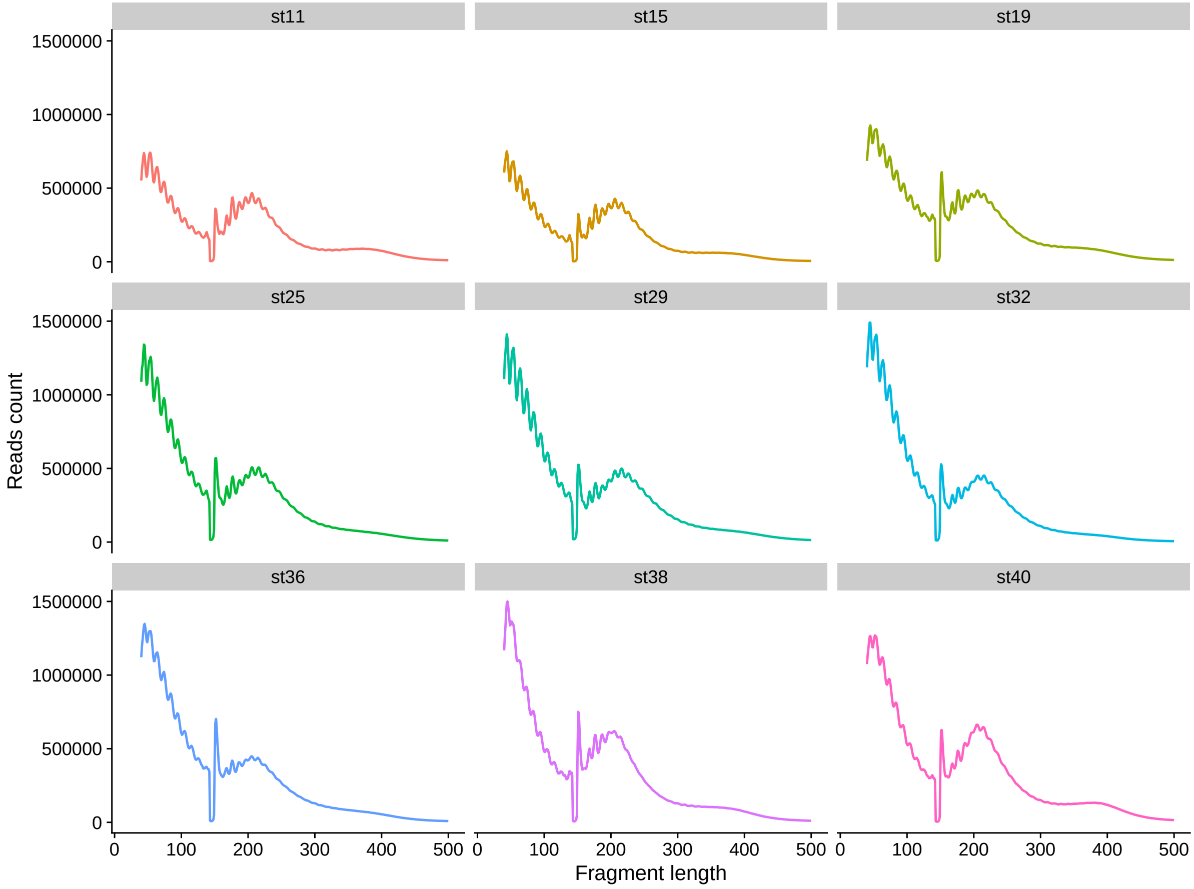

Supplementary Figures 13: ATAC-seq insert sizes determined by high-throughput sequencing. Adapters were not included and all fragments larger than 500 bp were removed.
