# Supplementary material for: Dynamic transcriptional and chromatin accessibility landscape of medaka embryogenesis
Source: Genome Res. 2020 Jun;30(6):924–37. doi: 10.1101/gr.258871.119 (PMC7370878; doi:10.1101/gr.258871.119)
Supplement: Supplemental Material [file supp_gr.258871.119_Supplemental_Fig_S14.pdf]

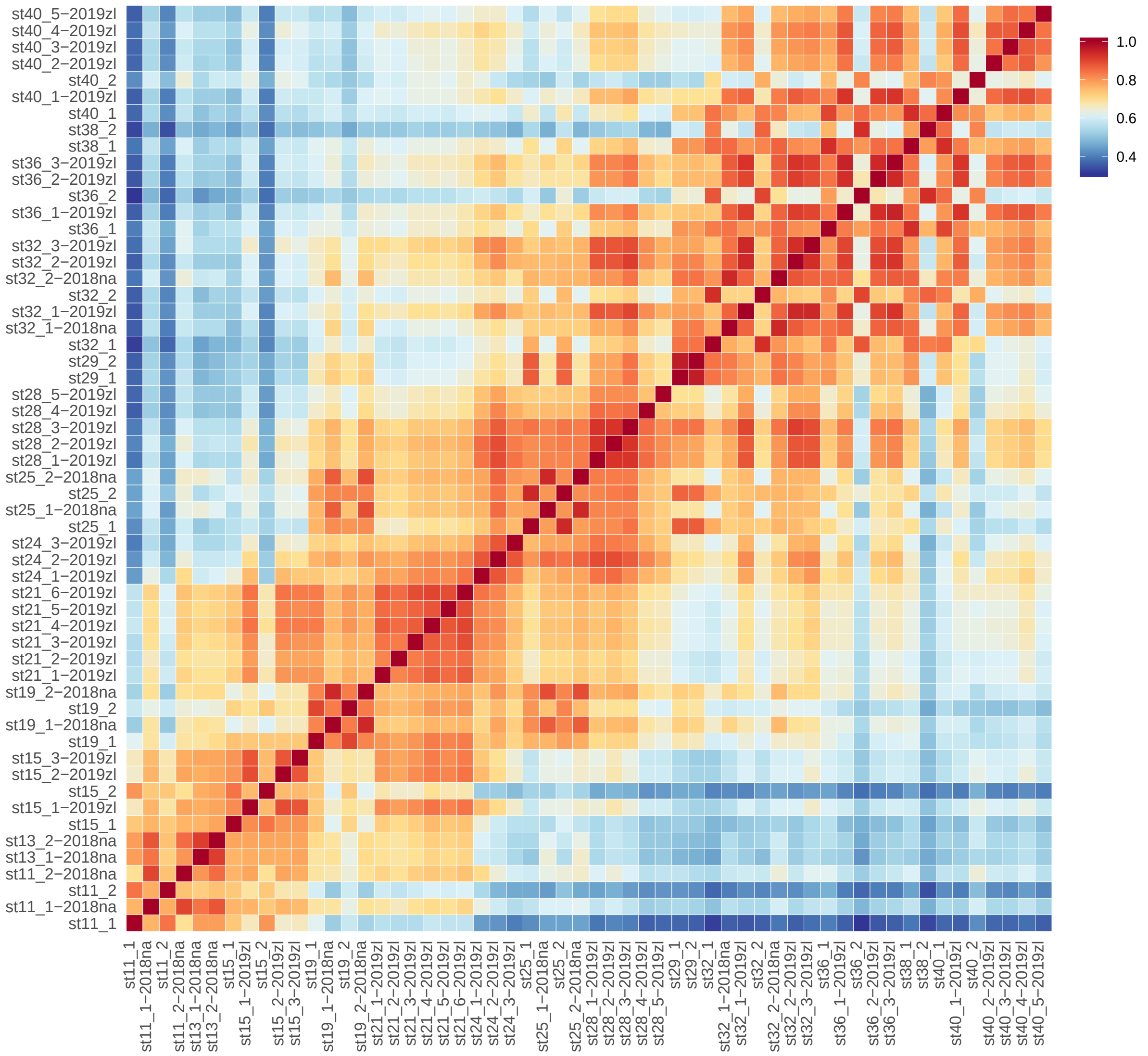

Supplementary Figures 14: Spearman's correlation among our ATAC-seq data and previously published data. All public samples are denoted by '2018na' or '2019zl'.
