# Supplementary material for: Dynamic transcriptional and chromatin accessibility landscape of medaka embryogenesis
Source: Genome Res. 2020 Jun;30(6):924–37. doi: 10.1101/gr.258871.119 (PMC7370878; doi:10.1101/gr.258871.119)
Supplement: Supplemental Material [file supp_gr.258871.119_Supplemental_Fig_S15.pdf]

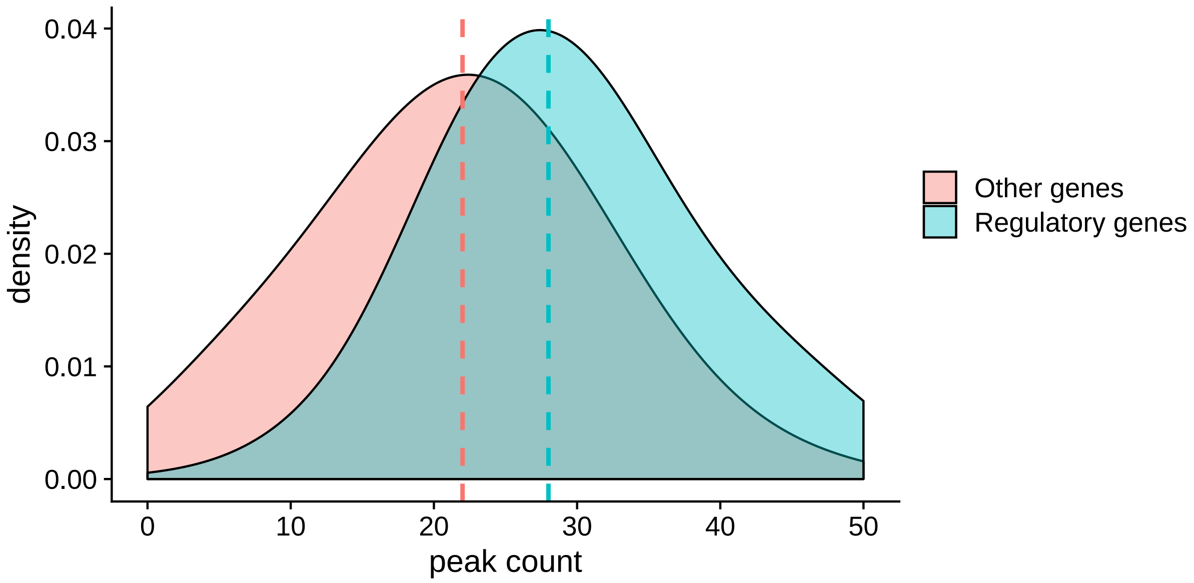

Supplementary Figures 15: Density plot of the number of accessible elements around regulatory genes or other genes (± 50kb). We calculated the number of accessible elements located within ±50 Kb of the TSS of each gene, and found that genomic regions adjacent to regulatory genes contain more accessible elements than regions adjacent to other genes do (median 22 versus 28).
