# Supplementary material for: Dynamic transcriptional and chromatin accessibility landscape of medaka embryogenesis
Source: Genome Res. 2020 Jun;30(6):924–37. doi: 10.1101/gr.258871.119 (PMC7370878; doi:10.1101/gr.258871.119)
Supplement: Supplemental Material [file supp_gr.258871.119_Supplemental_Fig_S18.pdf]

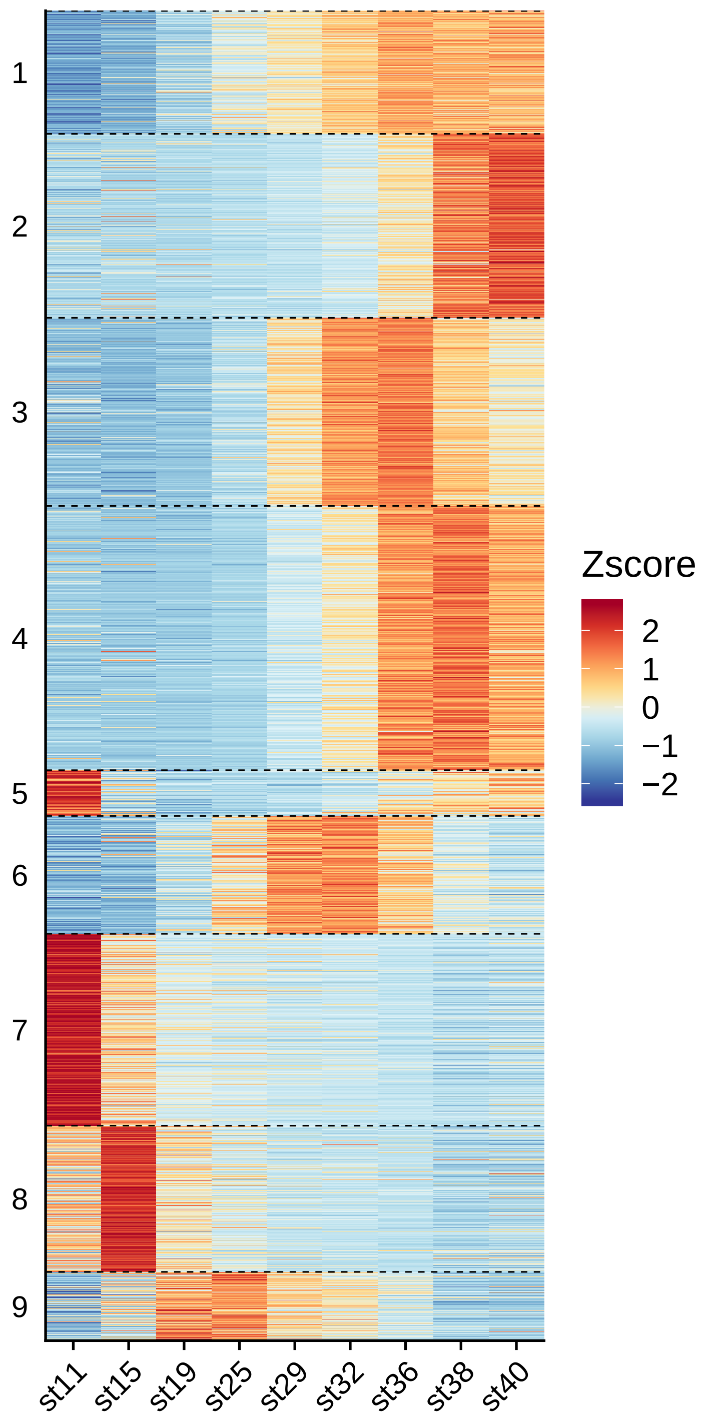

Supplementary Figures 18: K-means clustering of selected genes and gradually increased expression genes (in cluster 1, 2, 3, 4, 6, 8, and 9) were used to count early opening logic.
