# Supplementary material for: Dynamic transcriptional and chromatin accessibility landscape of medaka embryogenesis
Source: Genome Res. 2020 Jun;30(6):924–37. doi: 10.1101/gr.258871.119 (PMC7370878; doi:10.1101/gr.258871.119)
Supplement: Supplemental Material [file supp_gr.258871.119_Supplemental_Fig_S19.pdf]

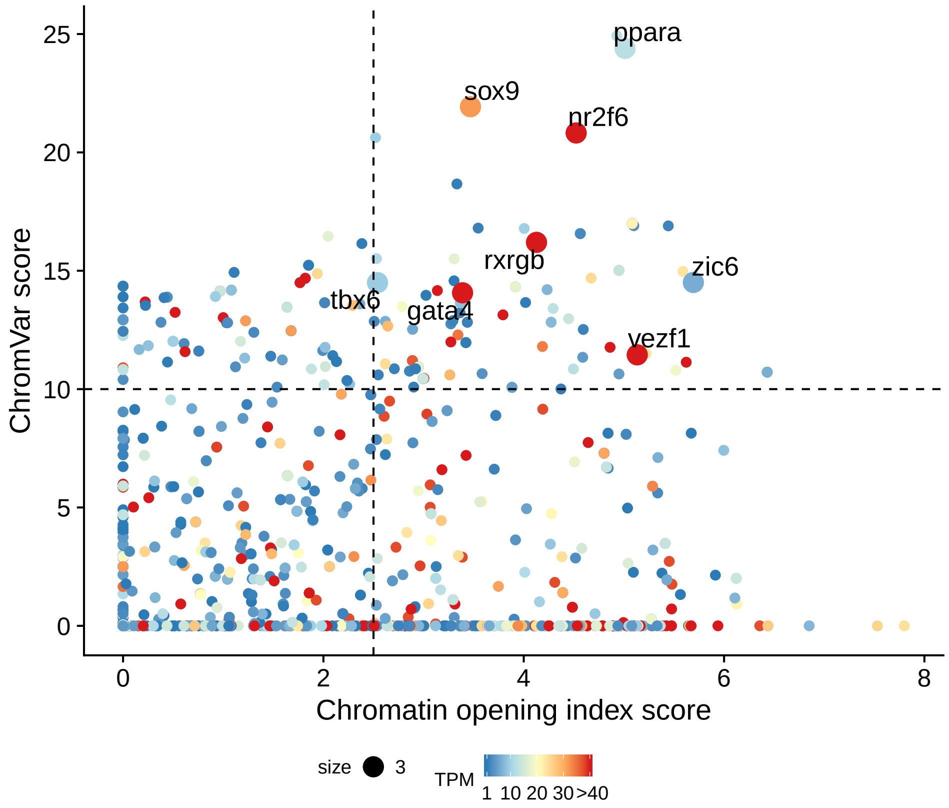

Supplementary Figures 19: Pioneer TF prediction at stage 19. Pioneer score is the x-axis, ChromVAR score is the y-axis, and the expression level is represented by the color. Pioneer TFs are those dots with high scores of three measurements, plotted with larger size.
