# Supplementary material for: Dynamic transcriptional and chromatin accessibility landscape of medaka embryogenesis
Source: Genome Res. 2020 Jun;30(6):924–37. doi: 10.1101/gr.258871.119 (PMC7370878; doi:10.1101/gr.258871.119)
Supplement: Supplemental Material [file supp_gr.258871.119_Supplemental_Fig_S20.pdf]

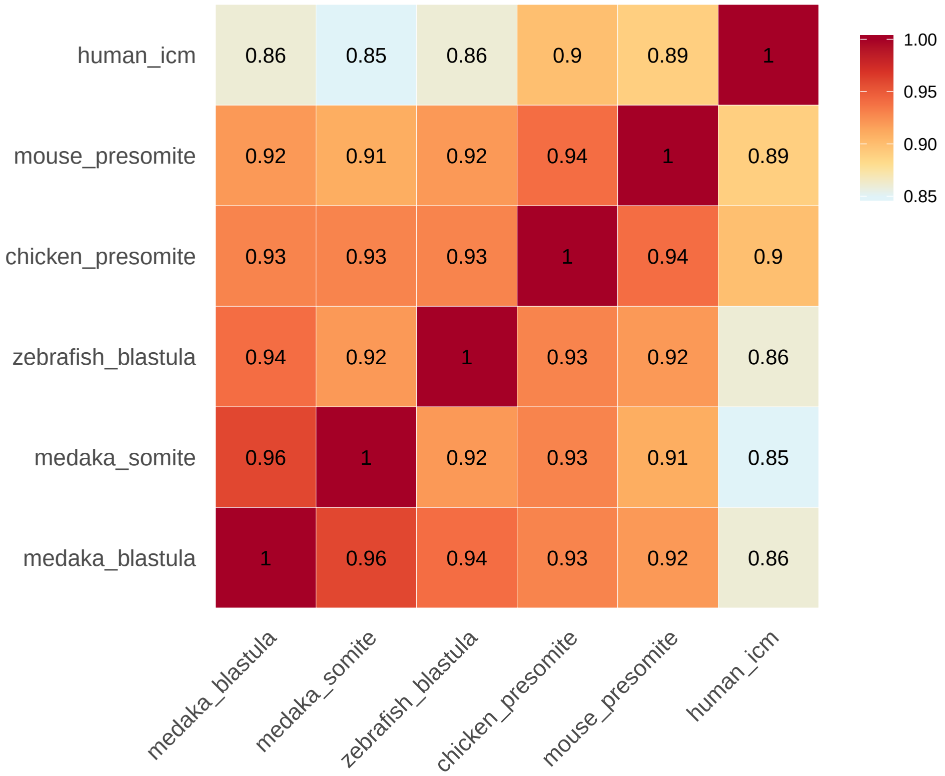

Supplementary Figures 20: Pioneer scores of medaka blastula (stage 11) , medaka somite (stage 19), zebrafish blastula (dome), chicken presomite (HH6), mouse presomite (E7.5) and human ICM are highly correlated.
