# Supplementary material for: Dynamic transcriptional and chromatin accessibility landscape of medaka embryogenesis
Source: Genome Res. 2020 Jun;30(6):924–37. doi: 10.1101/gr.258871.119 (PMC7370878; doi:10.1101/gr.258871.119)
Supplement: Supplemental Material [file supp_gr.258871.119_Supplemental_Fig_S21.pdf]

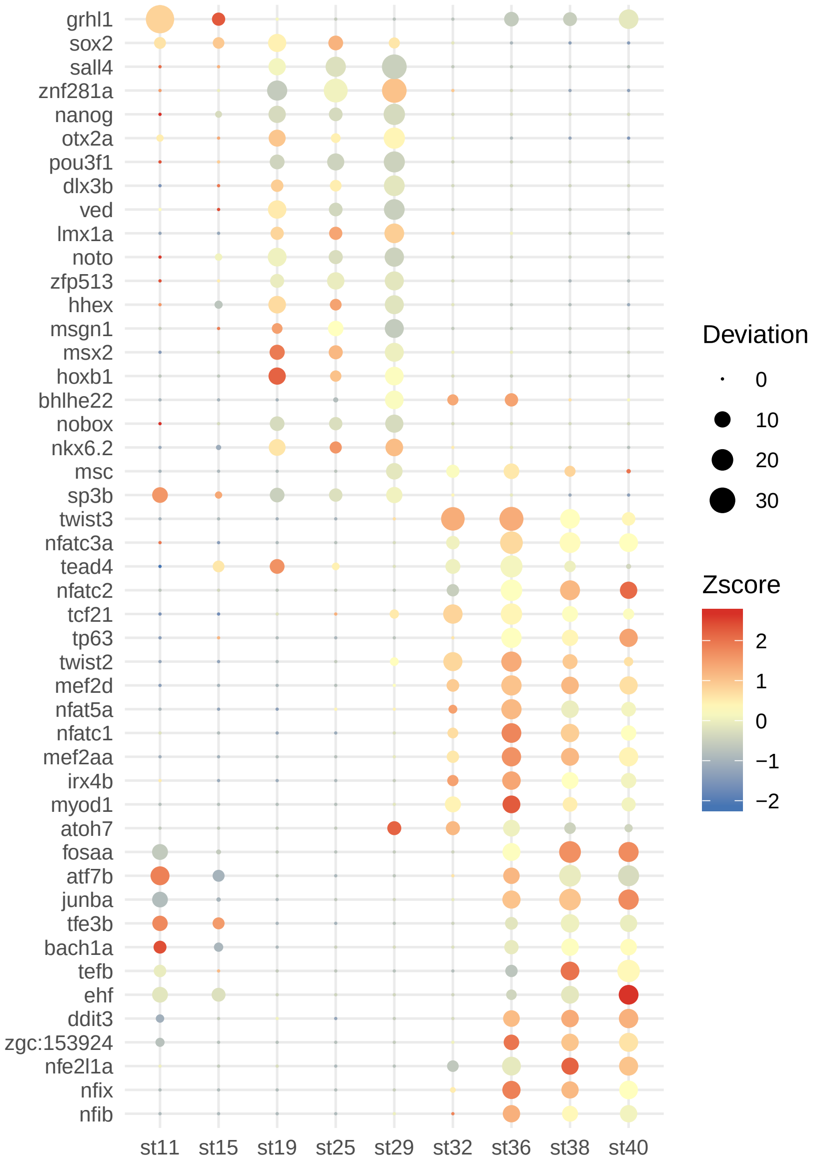

Supplementary Figures 21: TF expression and enrichment of its binding motif. The size of dots was correlated with accessible deviation calculated by ChromVAR, indicating the enrichment of TF motifs. The color of the dots reflects the expression level of the given TF.
