# Supplementary material for: Dynamic transcriptional and chromatin accessibility landscape of medaka embryogenesis
Source: Genome Res. 2020 Jun;30(6):924–37. doi: 10.1101/gr.258871.119 (PMC7370878; doi:10.1101/gr.258871.119)
Supplement: Supplemental Material [file supp_gr.258871.119_Supplemental_Fig_S22.pdf]

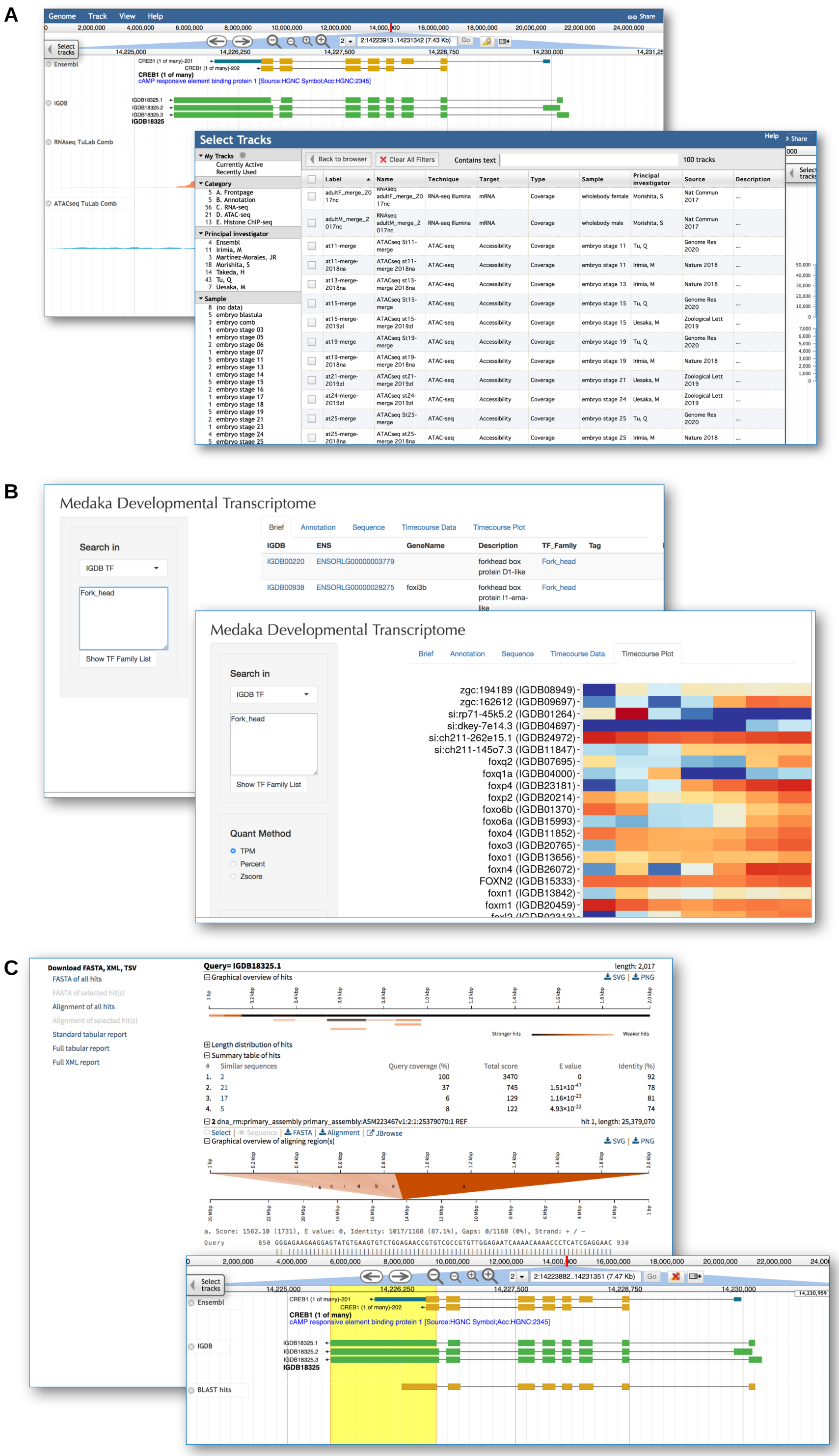

Supplementary Figures 22: Webtools for data query and visualization. (A) Genome browser, to search and visualize omics data of locus. (B) Gene viewer, to search and visualize gene annotation and expression data. (C) Sequence search (BLAST) tool, to search for similar sequences.
