# Supplementary material for: Dynamic transcriptional and chromatin accessibility landscape of medaka embryogenesis
Source: Genome Res. 2020 Jun;30(6):924–37. doi: 10.1101/gr.258871.119 (PMC7370878; doi:10.1101/gr.258871.119)
Supplement: Supplemental Material [file supp_gr.258871.119_Supplemental_Fig_S23.pdf]

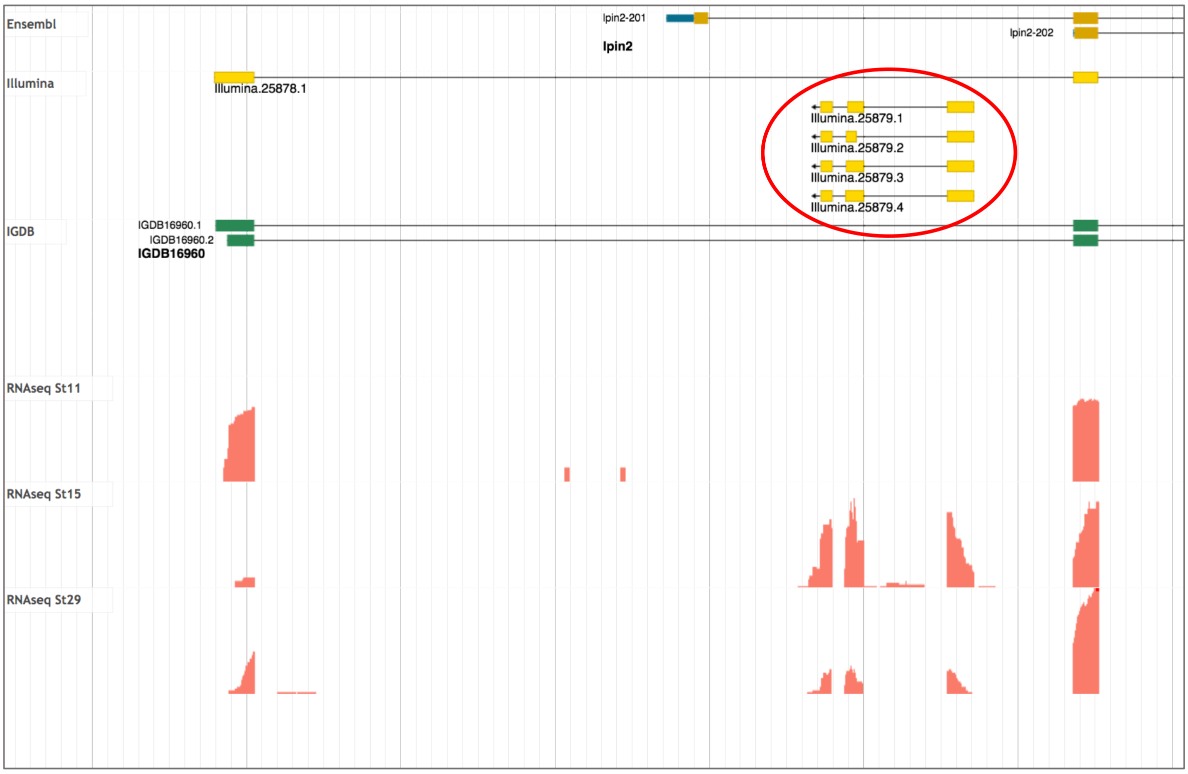

Supplementary Figures 23: LDAIR gene model. This lncRNA (red ellipse) was missed by the PacBio set, but was recovered from Illumina data. It is transiently expressed during stage 15 to 25.
