# Supplementary material for: Dynamic transcriptional and chromatin accessibility landscape of medaka embryogenesis
Source: Genome Res. 2020 Jun;30(6):924–37. doi: 10.1101/gr.258871.119 (PMC7370878; doi:10.1101/gr.258871.119)
Supplement: Supplemental Material [file supp_gr.258871.119_Supplemental_Methods_edited.pdf]

Dynamic Transcriptional and Chromatin Accessibility Landscape of Medaka Embryogenesis

Supplementary Methods

Yingshu Li[1,2,3,+], Yongjie Liu[1,2,3,+], Hang Yang[1,2,3], Ting Zhang[1,2], Kiyoshi Naruse[4,*], and Qiang Tu[1,2,3,*]

[1]*State Key Laboratory of Molecular Developmental Biology, Institute of Genetics and Developmental Biology, Innovation Academy for Seed Design, Chinese Academy of Sciences, Beijing 100101, China*
[2]*Key Laboratory of Genetic Network Biology, Institute of Genetics and Developmental Biology, Chinese Academy of Sciences, Beijing 100101, China.*
[3]*University of Chinese Academy of Sciences, Beijing 100049, China*
[4]*Laboratory of Bioresources, National Institute for Basic Biology, Okazaki 444-8585, Aichi, Japan*
[+]*These authors are joint first authors and contributed equally to this work.*
[*]*Corresponding authors: naruse@nibb.ac.jp, qtu@genetics.ac.cn*

Contents

1 Methods **2**
1.1 Sample collection . . . 2
1.2 RNA extraction . . . 2
1.3 PacBio long-read RNA sequencing . . . 2
1.4 Illumina short-read RNA sequencing . . . 2
1.5 Gene model construction and analysis . . . 3
1.6 ATAC-seq library preparation . . . 3
1.7 ATAC-seq sequencing . . . 4
1.8 ATAC-seq data analysis . . . 4

List of Figures

1 Methods

1.1 Sample collection

Embryonic developmental stages were determined according to the criteria set out by Iwamatsu (Iwamatsu, 2004). About 20-400 embryos were collected per stage per replicate depending on the RNA yield per embryo. Samples of 11 different developmental stages from stage 6 to stage 41 were collected from synchronous embryos, and we confirmed the diagnostic features one by one to ensure that all embryos were at the same developmental stage. For Illumina and PacBio sequencing, embryos were put in RNA later (Qiagen, cat. 76104) and stored at 4 °C no more than 1 week for RNA extraction. For ATAC-seq, embryos were collected in cold DPBS (Gibco, cat. C14190500BT) for embryo dissociation steps. We dissected adult medaka to isolate six different organ samples (brain, heart, ovary, testis, gut and muscle) using scissors and tweezers, and we mixed those six samples with equal RNA amount together for PacBio library construction and sequencing. Each type of adult organ comes from no fewer than six individuals. All samples, except gonads, contain equal number of male and female individuals, considering for the possible sex differences. Larval medaka gonads were obtained by microdissection, containing 30 male and female individuals separately.

1.2 RNA extraction

RNA was extracted using both TRIzol (Thermo Fisher Scientific, cat. 15596-018) and RNeasy Micro Kit (Qiagen, cat. 74004) with a modified protocol. Samples were first homogenized in TRIzol, then the phase separation step was performed according to the standard TRIzol protocol. Next, the aqueous phase containing RNA was transferred to the RNeasy Micro columns and processed following the RNeasy Micro protocol. In this way, complete lysis and high RNA yield from samples of different amounts were ensured. The RNA quality was assessed by Agilent Bioanalyzer 2100 system (Agilent Technologies). RNA samples with a RIN score of 9 or higher were used in further experiments.

1.3 PacBio long-read RNA sequencing

To avoid the loading bias of PacBio long-read libraries, which favors sequencing of shorter transcripts, multiple size-fractionated libraries (<5 kb and >5 kb) were constructed. Long reads/Barcoded SMRTBell libraries were sequenced on a PacBio Sequel platform by the commercial service provided by Annoroad Gene Technology Corporation (Beijing, China).

PacBio raw reads were first processed by the PacBio Iso-Seq pipeline (Gordon et al., 2015), which contains several steps: (1) getting Circular Consensus Sequences (CCS) reads out of subreads BAM file; (2) classifying reads into full-length or non-full-length reads, artificial-concatemer chimeric, or non-chimeric reads by identifying full-length CCS reads based on cDNA primers and polyA tail signals; (3) Cluster of FLNC reads using isoform-level clustering algorithm ICE (Iterative Clustering for Error Correction); (4) Polishing the full-length consensus sequences to generate high-quality (HQ), full-length, transcript isoform sequences.

1.4 Illumina short-read RNA sequencing

Stranded cDNA libraries of 13 samples (11 embryonic stages, adult ovary, and adult testis) were generated using TruSeq Stranded mRNA Library Prep (Illumina, cat. 20020594) and sequenced on the Illumina HiSeq X TEN platform (150-base paired-end) by the commercial service provided by Annoroad Gene Technology Corporation (Beijing, China).

Quality control was done using the FastQC package (version 0.11.3, `http://www.bioinformatics.babraham.ac.uk/projects/fastqc/`), the FASTX-Toolkit (version 0.0.14, `http://hannonlab.cshl.edu/fastx_toolkit/`) and the RSeQC package (version 2.6.6, `http://rseqc.sourceforge.net/`) (Wang et al., 2012). Reads were mapped to the medaka reference genome (Ichikawa et al., 2017) (`http://utgenome.org/medaka_v2/`) using Hisat2 (version 2.1.0) (Kim et al., 2015). Quantification of genes and isoforms was performed using StringTie (version v1.3.3b) (Pertea et al., 2015; Pertea et al., 2016) and the GTF annotation file of the IGDB set constructed in this study. Quantification was done for each replicate, and the TPM of each gene was averaged as the final quantification result, which was used for subsequent analysis.

1.5 Gene model construction and analysis

Mapping of PacBio HQ reads to the medaka reference genome was carried out using Spaln2 (Iwata and Gotoh, 2012). And the redundant transcripts model (GFF3 format) for each HQ long read from Spaln2 output were collapsed using the StringTie merge mode to generate a non-redundant set of transcript model (termed as PacBio set).

To assess the quality of the PacBio set, we calculated the short-read coverage of splice junctions observed in the PacBio set using QoRTs (version 1.3.6) (Hartley and Mullikin, 2015).

Each long-read isoform was compared with existing medaka Ensembl gene models (release 94, termed as Ensembl set) using gffcompare program (`https://ccb.jhu.edu/software/stringtie/gffcompare.shtml`), and the isoforms were further classified into seven groups based on their exon structures (splicing junctions). The gffcompare class codes used for long-read transcript classification in Figure 2B were: 'u' for 'Exact match', 'u' for 'Novel isoform from novel loci', 'e', 'j', and 'k' for 'Novel isoform from known loci', 'c' for 'Contained by reference', 'o' for 'Exonic overlap', 's' and 'x' for 'Exonic overlap on opposite strand', 'i', 'y', and 'p' for 'Other'. Also, both PacBio set and Ensembl set were compared with short-reads derived gene model (termed as Illumina set) to decide which model from the two sets is more reliable.

According to the gffcompare result of the PacBio set and Ensembl set, we classified the Ensembl transcripts into two class: Ensembl-only, which means an Ensembl transcript did not have any overlap with PacBio set models; and PacBio supported, which means an Ensembl transcript was supported by the PacBio set. We compared the expression level of these two classes of genes, using the maximum TPM obtained from the Illumina short-read data for all time-series samples.

We integrated the PacBio set and Ensembl set gene models into one set (termed as IGDB set). First, for the gene models with corresponding relationship (class code '=', 'c', 'j', 'e') between PacBio set and Ensembl set, we kept the gene models of the PacBio set; for the gene models unique to PacBio set or Ensembl set (class code 's', 'x', 'i', 'y', 'p', 'u'), we kept both PacBio set and Ensembl set models; for other models (class code 'k', 'o') that were difficult to decide, we compared them with Illumina set and kept the one more similar to Illumina set model.

For TF annotation, we generated the protein sequences of ORFs of the IGDB set using getorf (EMBOSS version 6.6.0.0), setting the minimum nucleotide size of ORF to 300. These sequences were screened using the TF prediction tool of AnimalTFDB 3.0 (Hu et al., 2019) to obtain the TF family annotation.

For lncRNA annotation, we first removed transcripts that are shorter than 200 nucleotides or have high sequence similarity to known medaka coding genes in Ensembl (BLAST *e*-value $< 10^{-50}$). Next, we scored the coding potential of sequences with the Coding Potential Assessment Tool (CPAT, version 1.2.2) (Wang et al., 2013) and the Coding Potential Calculator (CPC, version 2.0) software (Kang et al., 2017). CPAT scores were calculated using parameters '-d zebrafish_logitModel.RData -x zebrafish_Hexamer.tab' (Wang et al., 2013) and CPC scores were calculated using default parameters (Kang et al., 2017). Transcripts with high coding potential (>0.35 and >0.5 respectively) were removed.

Time course profiling data (TPM) were standardized using the z-score method. The Soft clustering analysis was carried out using the fuzzy c-means clustering implemented in the R Bioconductor package 'Mfuzz' (Kumar and Futschik, 2007), setting the fuzzifier (m) of 1.25 and the cluster number (c) of 30, and select the clustering result with a membership value larger than 0.3 for further analysis. GO term enrichment analysis of clusters from Mfuzz was performed with a focus on biological processes using the R Bioconductor package clusterProfiler (Yu et al., 2012) (https://www.bioconductor.org/packages/clusterProfiler), setting the q-value threshold as 0.05 for statistical significance. We also generated bubble plots of significant results for each cluster using ClusterProfiler.

1.6 ATAC-seq library preparation

ATAC-seq libraries were prepared as previously reported (Corces et al., 2017; Wu et al., 2018) with the some modifications. Cell pellets were resuspended in 50 µl cold ATAC-Resuspension Buffer (RSB) containing 0.1% NP40, 0.1% Tween-20, and 0.01% digitonin and incubate on ice for 3 minutes. After lysis, 1 ml of ATAC-seq RSB containing 0.1% Tween-20 (without NP40 or digitonin) was added, and the tubes were inverted to mix. Nuclei were then centrifuged for 10 min at 500 r.c.f. in a pre-chilled (4 °C) fixed-angle centrifuge. Supernatant was removed and nuclei were resuspended in 50 µl of transposition

mix (10 µl 5× TTBL, 5 µl TTEmix V50 (TD501, Vazyme), 0.5 µl 1% digitonin, 0.5 µl 10% Tween-20, 34 µl ddH_2O). Transposition reactions were incubated at 37 °C for 30 min with shaking at 1,000 r.p.m. After add 2 µl carrier RNA (20 ng/µl after 50× dilution), 78 µl Tris-EDTA (TE) buffer and 130 µl phenol-chloroform (vortexed and incubated at room temperature for 3 min) to the transposition reactions, the sample was transferred to a phase-lock tube (WM5-2302820 TIANGEN) and spinning at maximum speed for 15 min. The supernatant was transferred to a new 1.5-ml tube, and 650 µl ethanol, 24 µl sodium acetate (3 M) and 2 µl glycogen (Invitrogen UltraPure Glycogen) were added for DNA precipitation at −20 °C overnight. The next day, the DNA pellet was spun down at maximum speed for 15 min at 4 °C, washed with 75% ethanol, air dried, and resuspended in 24 µl ddH_2O). DNA was transferred to a 0.2-ml PCR tube, and 5 µl N5XX primer, 5 µl N7XX primer (TD202, Vazyme), 5 µl PPM, 10 µl 5× TAB and 1 µl TAE (TD501, Vazyme) were added. PCR was performed to amplify the library for 8-10 cycles using the following PCR conditions: 72 °C for 3 min; 98 °C for 30 s; and thermocycling at 98 °C for 15 s, 60 °C for 30 s and 72 °C for 3 min; following by 72 °C 5 min. Follow the PCR reaction, 0.6 × AMPure (Beckman) beads were used to remove large fragments and 1.2× AMPure (Beckman) to recover the library.

1.7 ATAC-seq sequencing

All ATAC-seq libraries were sequenced on the Illumina HiSeq X TEN platform (150-base paired-end) by the commercial service provided by Annoroad Gene Technology Corporation (Beijing, China). Reads were trimmed using Trimmomatic (version 0.36) (Bolger et al., 2014), and then aligned to the medaka reference genome (Ichikawa et al., 2017) by Bowtie (version 2.3.4.1) (Langmead and Salzberg, 2012) with parameters '-X 2000 –very-sensitive –qc-filter'. All unmapped reads, non-uniquely mapped reads, mitochondria reads and large inserted fragment(> 150 bp) were removed by samtools (version 1.9) (Li et al., 2009). Then, PCR duplicates reads were removed using Picard (`http://broadinstitute.github.io/picard/`). All peak calling was performed with MACS2 (version 2.1.2) (Zhang et al., 2008) using parameters 'macs2 callpeak -f BAMPE –keep-dup all -g 7.34e8'. Peaks from all stages were filted with a stringent cutoff (-log(q-value) > 7), then merged and peak reads counts were calculated by the multicov function from BEDTools (Quinlan, 2014). For downstream analysis, first merge replicates of each developmental stages and normalized peak reads by computing numbers of peak reads per million of peak reads. To visualize the ATAC-seq signal in the JBrowse and minimize the batch, we counted the coverage for each base with deeptools (Ramírez et al., 2016) using 'deeptools bamCoverage –binSize 1 –extendReads –normalizeUsing CPM'.

1.8 ATAC-seq data analysis

Accessible elements distribution were calculated according to their genomic location: promoters, within 2 kb upstream and 0.5 kb downstream of a TSS; gene body, within a gene; proximal, between 2 kb to 5 kb upstream of a TSS; distal, not in any categories described above.

ATAC-seq data for multiple species analysis are collected: SRR5860468,SRR5860469 and SRR5860470 for zebrafish dome stage ; SRR6940543, SRR6940542, SRR6940541, SRR6940540, SRR5837341, SRR5837340, SRR5837339, SRR5837338 SRR5837337 for human ICM ; DRR138947, DRR138948, DRR138949 for chicken presomite stage ; DRR138923, DRR138924, DRR138925 for mouse presomite stage. And the analysis pipeline is similar with our data.

To examine the selective constraint of accessible elements, medaka conserved regions among 51 fish genome was download from Ensembl Compara (`http://ftp.ensemblorg.ebi.ac.uk/pub/release-97/bed/ensembl-compara/51_fish.gerp_constrained_element/gerp_constrained_elements.oryzias_latipes.bb`). Constraint scores (i.e. rejected substitutions scores) of individual regions were calculated with GERP++ (Davydov et al., 2010) and 51 fish genomes, indicating the sequence conservation among these fish genomes. All of regions shorter than 10 nucleotides were removed and merged with accessible elements using 'bedtools intersect -wa -wb -sorted'. Accessible elements that overlapped with constraint regions was defined as conserved. RS score of each conserved accessible element was represented by the maximum RS scores of all overlapped constraint regions.

To consider generally how common accessible elements were open earlier than the corresponding gene started to express, pairwise Spearman's correlations between accessibile elements read counts and mRNA expression of the gene across the embryogenesis were evaluated. A null distribution of Spearman's correlation value was estimated empirically, by pairing the promoters elements with 2000 randomly picked

genes. After that, the z-score for the correlation coefficient was then calculated by subtracting the mean and dividing by the standard deviation estimated from the empirical null. The corresponding P-value was calculated using the pnorm function in R. Only those genes showing a P-value 0.1 (Spearman's correlation coefficient bigger than 0.7) were retained. We then clustered all these genes using K-means and genes with gradually increased expression were used to count early opening logic (Supplemental Fig. 15).

TF binding motif enrichment was calculated using chromVAR (Schep et al., 2017). We first forged a BSgenome package for *Oryzias latipes*. We utilized chromVAR with default parameters according to a standard walkthrough. For motif matching, we collected human, mouse, zebrafish, and medaka motifs from the Cis-BP database (build 2.00) (Weirauch et al., 2014; Lambert et al., 2019). For all motifs, frequencies were first normalized by the LICORS package with tolerance level 10^{-6}. Frequencies were then re-normalized to sum to 1, before a 0.008 pseudo count was added.

Chromatin opening index scores of multiple species was calculated using Protein Interaction Quantification (PIQ) (Sherwood et al., 2014).The source code for PIQ was modified to run with related genome (human hg19, mouse GRCm38, chicken GRCg6a, zebrafish GRCz11 and medaka ASM223467v1). To get a panoramic chromatin opening index score, PIQ was run according to default parameters using default JASPAR motifs (Fornes et al., 2020) and Cis-BP motifs as previse described. Spearman's correlations of chromatin opening index scores of motifs among those species were caculated.

References

Bolger AM, Lohse M, and Usadel B. 2014. Trimmomatic: A Flexible Trimmer for Illumina Sequence Data. *Bioinformatics.* **30**: 2114–2120.

Corces MR, Trevino AE, Hamilton EG, Greenside PG, Sinnott-Armstrong NA, Vesuna S, Satpathy AT, Rubin AJ, Montine KS, Wu B, et al. 2017. An Improved ATAC-Seq Protocol Reduces Background and Enables Interrogation of Frozen Tissues. *Nat. Methods.* **14**: 959–962.

Davydov EV, Goode DL, Sirota M, Cooper GM, Sidow A, and Batzoglou S. 2010. Identifying a High Fraction of the Human Genome to Be under Selective Constraint Using GERP++. *PLoS Comput. Biol.* **6**: e1001025.

Fornes O, Castro-Mondragon JA, Khan A, van der Lee R, Zhang X, Richmond PA, Modi BP, Correard S, Gheorghe M, Baranašić D, et al. 2020. JASPAR 2020: Update of the Open-Access Database of Transcription Factor Binding Profiles. *Nucleic Acids Res.* **48**: D87–D92.

Gordon SP, Tseng E, Salamov A, Zhang J, Meng X, Zhao Z, Kang D, Underwood J, Grigoriev IV, Figueroa M, et al. 2015. Widespread Polycistronic Transcripts in Fungi Revealed by Single-Molecule mRNA Sequencing. *PLoS ONE.* **10**: e0132628.

Hartley SW and Mullikin JC. 2015. QoRTs: A Comprehensive Toolset for Quality Control and Data Processing of RNA-Seq Experiments. *BMC Bioinformatics.* **16**: 224.

Hu H, Miao YR, Jia LH, Yu QY, Zhang Q, and Guo AY. 2019. AnimalTFDB 3.0: A Comprehensive Resource for Annotation and Prediction of Animal Transcription Factors. *Nucleic Acids Res.* **47**: D33–D38.

Ichikawa K, Tomioka S, Suzuki Y, Nakamura R, Doi K, Yoshimura J, Kumagai M, Inoue Y, Uchida Y, Irie N, et al. 2017. Centromere Evolution and CpG Methylation during Vertebrate Speciation. *Nat Commun.* **8**: 1833.

Iwamatsu T. 2004. Stages of Normal Development in the Medaka Oryzias Latipes. *Mech. Dev.* **121**: 605–618.

Iwata H and Gotoh O. 2012. Benchmarking Spliced Alignment Programs Including Spaln2, an Extended Version of Spaln That Incorporates Additional Species-Specific Features. *Nucleic Acids Res.* **40**: e161.

Kang YJ, Yang DC, Kong L, Hou M, Meng YQ, Wei L, and Gao G. 2017. CPC2: A Fast and Accurate Coding Potential Calculator Based on Sequence Intrinsic Features. *Nucleic Acids Res.* **45**: W12–W16.

Kim D, Langmead B, and Salzberg SL. 2015. HISAT: A Fast Spliced Aligner with Low Memory Requirements. *Nat. Methods.* **12**: 357–360.

Kumar L and Futschik M. 2007. Mfuzz: A Software Package for Soft Clustering of Microarray Data. *Bioinformation.* **2**: 5–7.

Lambert SA, Yang AWH, Sasse A, Cowley G, Albu M, Caddick MX, Morris QD, Weirauch MT, and Hughes TR. 2019. Similarity Regression Predicts Evolution of Transcription Factor Sequence Specificity. *Nat. Genet.* **51**: 981–989.

Langmead B and Salzberg SL. 2012. Fast Gapped-Read Alignment with Bowtie 2. *Nat. Methods.* **9**: 357–359.

Li H, Handsaker B, Wysoker A, Fennell T, Ruan J, Homer N, Marth G, Abecasis G, Durbin R, and 1000 Genome Project Data Processing Subgroup. 2009. The Sequence Alignment/Map Format and SAMtools. *Bioinformatics.* **25**: 2078–2079.

Pertea M, Pertea GM, Antonescu CM, Chang TC, Mendell JT, and Salzberg SL. 2015. StringTie Enables Improved Reconstruction of a Transcriptome from RNA-Seq Reads. *Nat. Biotechnol.* **33**: 290–295.

Pertea M, Kim D, Pertea GM, Leek JT, and Salzberg SL. 2016. Transcript-Level Expression Analysis of RNA-Seq Experiments with HISAT, StringTie and Ballgown. *Nat Protoc.* **11**: 1650–1667.

Quinlan AR. 2014. BEDTools: The Swiss-Army Tool for Genome Feature Analysis. *Curr Protoc Bioinformatics.* **47**: 11.12.1–34.

Ramírez F, Ryan DP, Grüning B, Bhardwaj V, Kilpert F, Richter AS, Heyne S, Dündar F, and Manke T. 2016. deepTools2: A next Generation Web Server for Deep-Sequencing Data Analysis. *Nucleic Acids Res.* **44**: W160–165.

Schep AN, Wu B, Buenrostro JD, and Greenleaf WJ. 2017. chromVAR: Inferring Transcription-Factor-Associated Accessibility from Single-Cell Epigenomic Data. *Nat. Methods.* **14**: 975–978.

Sherwood RI, Hashimoto T, O'Donnell CW, Lewis S, Barkal AA, van Hoff JP, Karun V, Jaakkola T, and Gifford DK. 2014. Discovery of Directional and Nondirectional Pioneer Transcription Factors by Modeling DNase Profile Magnitude and Shape. *Nat. Biotechnol.* **32**: 171–178.

Wang L, Wang S, and Li W. 2012. RSeQC: Quality Control of RNA-Seq Experiments. *Bioinformatics.* **28**: 2184–2185.

Wang L, Park HJ, Dasari S, Wang S, Kocher JP, and Li W. 2013. CPAT: Coding-Potential Assessment Tool Using an Alignment-Free Logistic Regression Model. *Nucleic Acids Res.* **41**: e74.

Weirauch MT, Yang A, Albu M, Cote AG, Montenegro-Montero A, Drewe P, Najafabadi HS, Lambert SA, Mann I, Cook K, et al. 2014. Determination and Inference of Eukaryotic Transcription Factor Sequence Specificity. *Cell.* **158**: 1431–1443.

Wu J, Xu J, Liu B, Yao G, Wang P, Lin Z, Huang B, Wang X, Li T, Shi S, et al. 2018. Chromatin Analysis in Human Early Development Reveals Epigenetic Transition during ZGA. *Nature.* **557**: 256–260.

Yu G, Wang LG, Han Y, and He QY. 2012. clusterProfiler: An R Package for Comparing Biological Themes among Gene Clusters. *OMICS.* **16**: 284–287.

Zhang Y, Liu T, Meyer CA, Eeckhoute J, Johnson DS, Bernstein BE, Nusbaum C, Myers RM, Brown M, Li W, et al. 2008. Model-Based Analysis of ChIP-Seq (MACS). *Genome Biol.* **9**: R137.
